# Pathways of Carbon Assimilation and Ammonia Oxidation Suggested by Environmental Genomic Analyses of Marine *Crenarchaeota*

**DOI:** 10.1371/journal.pbio.0040095

**Published:** 2006-03-21

**Authors:** Steven J Hallam, Tracy J Mincer, Christa Schleper, Christina M Preston, Katie Roberts, Paul M Richardson, Edward F DeLong

**Affiliations:** **1**Massachusetts Institute of Technology, Cambridge, Massachusetts, United States of America; **2**Department of Biology, University of Bergen, Bergen, Norway; **3**Monterey Bay Aquarium Research Institute, Moss Landing, California, United States of America; **4**Department of Geological & Environmental Sciences, Stanford University, Stanford, California, United States of America; **5**Joint Genome Institute, Walnut Creek, California, United States of America; Woods Hole Oceanographic InstitutionUnited States of America

## Abstract

Marine *Crenarchaeota* represent an abundant component of oceanic microbiota with potential to significantly influence biogeochemical cycling in marine ecosystems. Prior studies using specific archaeal lipid biomarkers and isotopic analyses indicated that planktonic *Crenarchaeota* have the capacity for autotrophic growth, and more recent cultivation studies support an ammonia-based chemolithoautotrophic energy metabolism. We report here analysis of fosmid sequences derived from the uncultivated marine crenarchaeote, *Cenarchaeum symbiosum,* focused on the reconstruction of carbon and energy metabolism. Genes predicted to encode multiple components of a modified 3-hydroxypropionate cycle of autotrophic carbon assimilation were identified, consistent with utilization of carbon dioxide as a carbon source. Additionally, genes predicted to encode a near complete oxidative tricarboxylic acid cycle were also identified, consistent with the consumption of organic carbon and in the production of intermediates for amino acid and cofactor biosynthesis. Therefore, C. symbiosum has the potential to function either as a strict autotroph, or as a mixotroph utilizing both carbon dioxide and organic material as carbon sources. From the standpoint of energy metabolism, genes predicted to encode ammonia monooxygenase subunits, ammonia permease, urease, and urea transporters were identified, consistent with the use of reduced nitrogen compounds as energy sources fueling autotrophic metabolism. Homologues of these genes, recovered from ocean waters worldwide, demonstrate the conservation and ubiquity of crenarchaeal pathways for carbon assimilation and ammonia oxidation. These findings further substantiate the likely global metabolic importance of *Crenarchaeota* with respect to key steps in the biogeochemical transformation of carbon and nitrogen in marine ecosystems.

## Abstract 2

Sequence data reveal the presence of key genes from pathways for carbon assimilation and ammonia oxidation in marine microbiota, supporting their importance in regulating the biogeochemistry of marine ecosystems.

## Introduction

*Crenarchaeota* are abundant in the world's oceans, comprising an estimated 20% of all planktonic prokaryotes [ 1, 2]. They are distributed over a wide depth range, spanning both euphotic and aphotic zones [ 3– 5], and at least one species, *Cenarchaeum symbiosum,* has a symbiotic association with the marine sponge Axinella mexicana [ 6]. Given their numerical abundance and cosmopolitan nature, *Crenarchaeota* represent an important constituent of marine ecosystems around the globe.

Organic geochemical biomarkers in the form of lipid biomass have proven successful for tracking *Archaea* in marine sediments and in the water column. Unlike the fatty acid–derived membranes of bacteria and eukarya, the major components of archaeal cell membranes are isoprenoid ether-linked glyceroldiethers or tetraethers [ 7, 8]. Planktonic *Archaea* produce glycerol dialkyl glycerol tetraethers [ 9– 11], including one unique structure crenarchaeol [ 7, 12, 13] often considered a diagnostic biomarker for planktonic archaeal species. It remains unclear if this compound is unique to marine *Crenarchaeota,* or is found in both euryarchaeotal lineages, too. Radiocarbon analyses of ^14^C in lipid biomarkers associated with marine plankton [ 14], and ^13^C-labeled bicarbonate tracer studies [ 15] suggest that marine *Crenarchaeota* are capable of light-independent autotrophic carbon assimilation into membrane lipid biomass, an hypothesis further strengthened by recent single cell phylogenetic identification and autoradiographic verification of carbon dioxide incorporation [ 16]. The direct incorporation of dissolved inorganic carbon by marine *Crenarchaeota* may be homologous to metabolic properties of more distantly related thermophilic *Crenarchaeota* that utilize a modified 3-hydroxypropionate [ 17– 21] or reductive tricarboxylic acid [ 22– 24] cycle for autotrophic carbon assimilation.

Assuming marine *Crenarchaeota* do assimilate carbon autotrophically, the energy source utilized for growth must be consistent with the surrounding chemical environment. Ammonia (NH _3_) produced by the decomposition of organic matter has the potential to provide both nutrient nitrogen and a source of reducing power. The seasonal distribution of marine *Crenarchaeota* in the oxic and ammonia-rich surface waters off Palmer Station, Antarctica [ 4], as well as a correlation of increasing crenarchaeal abundance with a nitrite (NO _2_^−^) maximum are both consistent with the hypothesis that marine *Crenarchaeota* are capable of ammonia oxidation [ 25]. Whole genome shotgun (WGS) analysis of DNA sequences derived from the Sargasso Sea (SAR) identified potential ammonia monooxygenase *(amo)* genes associated with presumptive archaeal contigs supporting this hypothesis [ 26]. Another study of fosmids derived from complex soil libraries identified *amo* gene sequences related to those observed in the SAR, linked to a crenarchaeal ribosomal RNA operon, and a recent PCR survey has confirmed the widespread occurance of archaeal *amoA* genes in marine water columns and sediments [ 27], reinforcing a potential and widespread role for ammonia oxidation in varied archaeal lineages [ 28]. Recent isolation of a marine crenarchaeote in pure culture using bicarbonate and ammonia as sole carbon and energy sources strongly supports this hypothesis [ 29]. However, the specific biochemical pathways mediating ammonia oxidation and carbon assimilation in this isolate remain unknown.

*C. symbiosum,* a mesophilic crenarchaeal symbiont of the marine sponge Axinella mexicana [ 6] provides a useful system for modeling the physiology and genetics of marine *Crenarchaeota*. Previous studies have determined that C. symbiosum constitutes the sole archaeal phylotype associated with *A. mexicana,* reaching up to 65% of the total prokaryotic cell population within a given host [ 6]. The purity and abundance of C. symbiosum cells in host tissue has enabled the construction of fosmid DNA libraries containing its full genomic repertoire ([ 30, 31] and Hallam et al., unpublished data). Although C. symbiosum is a sponge symbiont and therefore at some level adapted to life within its host environment, it remains phylogenetically clustered with the planktonic *Crenarchaeota* [ 6]. Given this relationship, sequence data obtained from C. symbiosum genomic libraries can be used to query and interpret crenarchaeal sequences recovered from the planktonic environment [ 32, 33]. We report here analyses of unassembled C. symbiosum genomic DNA sequences, combined with environmental gene and database surveys, to determine the presence and distribution of highly conserved genes with the potential to mediate carbon assimilation and ammonia oxidation in marine *Crenarchaeota*.

## Results

### Environmental Genomic Analysis of a Fosmid Library Enriched for C. symbiosum DNA

C. symbiosum cells were enriched from host tissue using differential density centrifugation (see Materials and Methods). High molecular weight DNA purified from this cell enrichment was used to construct two 32–45 Kb insert fosmid DNA libraries composed of 10,236 and 2,100 clones, respectively [ 31]. Seven C. symbiosum SSU rRNA genes were identified in the first library, representing approximately 0.07% of the total clone population. Eight C. symbiosum SSU rRNA genes were identified in the second library, representing approximately 0.38% of the total clone population [ 31]. For a 2 million base pair (Mb) genome containing one copy of the SSU rRNA gene, approximately 50 fosmids arranged in a linear tiling path have the potential to cover the entire genomic sequence. Given this estimate and the percentage of C. symbiosum SSU rRNA genes identified in the second library, approximately 400 of the 2,100 clones in the library should be derived from C. symbiosum donors (˜8-fold coverage of a 2-Mb genome). Paired-end sequencing of the smaller fosmid library generated 1.8 Mb of DNA sequence from 2,779 nonredundant reads greater than 200 bp (base pair) per read. A total of 168 fosmids encompassing more than 6.4 Mb of genomic DNA were selected for subcloning and sequencing based on the following criteria: (1) paired ends predicted to contain open reading frames most similar to archaeal genes, (2) linkage with previously reported fosmids harboring crenarchaeal phylogenetic anchors, and (3) sets of paired ends, assembled in opposing orientations and predicted to contain open reading frames homologous to two or more archaeal genes. On average, selected fosmids had a G+C content of 58%. In general, the identity of completed fosmids could be verified on the combined basis of G+C content, SSU rRNA, or functional gene linkage, and the taxonomic distribution of predicted open reading frames contained on individual fosmid sequences. Based on this set of criteria, 155 of the completed fosmids were derived from *C. symbiosum,* ten from unspecified bacteria and three from host donors.

Overall genomic representation of the dataset was determined based on the identification of complete or redundant sets of genes encoding ribosomal proteins, amino-acyl tRNA synthases, and conserved components of several core processes including but not limited to the transcription, translation, and replication machinery identified within the set of 155 archaeal fosmids. On average, two copies of each gene within a given category were identified (unpublished data), consistent with 2- to 3-fold coverage of an estimated 2-Mb genome. Previous studies of C. symbiosum population structure identified two coexisting ribotypic variants, a and b, exhibiting approximately 99% nucleotide identity at the ribosomal level but ranging between 70% and 90% nucleotide identity in genomic intervals adjacent to the ribosomal operon [ 30]. Despite this variation in nucleotide identity, gene content and order appeared to be conserved between the two ribotypes [ 30]. The present study focuses on pathways of autotrophic carbon assimilation and ammonia oxidation, based on analyses of individual fosmid sequences, and does not attempt to discriminate between sequences derived from a or btypes. The issue of heterogeneity and the assembly of a-type and b-type genomic scaffolds will be the subject of future work exploring the complete genome sequence of C. symbiosum (Hallam et al., unpublished data).

### Autotrophic Carbon Assimilation Genes in C. symbiosum

In order to identify potential effectors of autotrophic metabolism in the unassembled C. symbiosum genomic sequences, systematic searches for each of the four known pathways of autotrophic CO _2_ fixation were conducted (see Materials and Methods). These include: (1) the unidirectional reductive pentose phosphate cycle [ 34], (2) the bidirectional reductive acetyl coenzyme A (CoA) pathway [ 35], the (3) the reductive tricarboxylic acid pathway [ 36], and (4) the unidirectional 3-hydroxypropionate cycle [ 37]. Each pathway is defined by a subset of diagnostic enzymes. The reductive pentose phosphate cycle requires the activities of ribulose 1,5-bisphosphate carboxylase, phosphoribulokinase, and sedoheptulose bisphosphatase [ 38]. The reductive acetyl-CoA pathway requires the activity of carbon monoxide dehydrogenase [ 39]. The reductive TCA (tricarboxylic acid) cycle requires the activities of citrate lyase, 2-oxoglutarate:ferredoxin oxidoreductase, and fumarate reductase [ 40]. Finally, the 3-hydroxypropionate cycle requires the activities of acetyl-CoA/propionyl-CoA carboxylase [ 37], malonyl-CoA reductase [ 41], and propionyl-CoA synthase [ 42]. Search results identified numerous components of the 3-hydroxypropionate cycle and the citric acid cycles ( Tables 1, 2, and S1). Homologues for 1,5-bisphosphate carboxylase/oxygenase (RubisCO), phosphoribulokinase, sedoheptulose bisphosphatase, and carbon monoxide dehydrogenase representing the reductive pentose phosphate cycle and reductive acetyl-CoA cycle, respectively, were not identified.

**Table 1:**
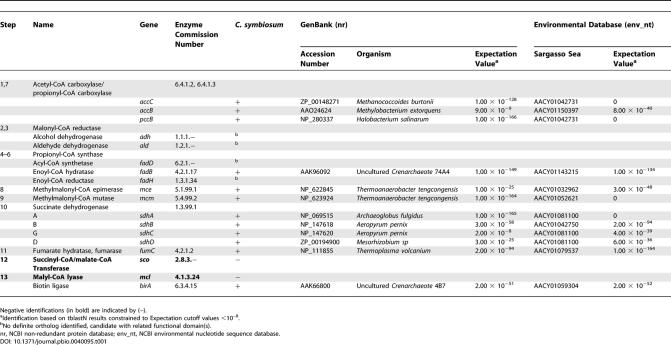
3-Hydroxypropionate Cycle Components Identified on C. symbiosum Fosmids

**Table 2:**
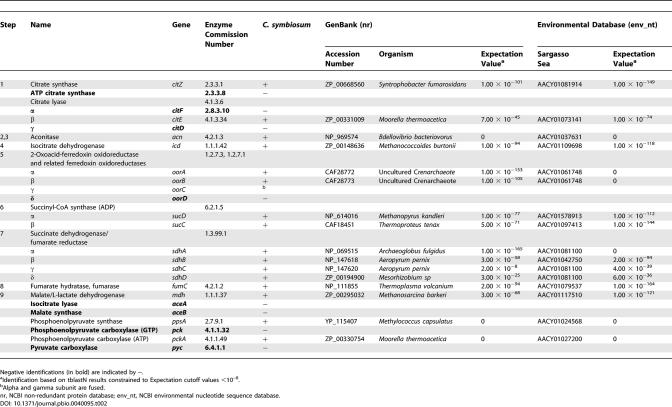
TCA Cycle Components Identified on C. symbiosum Fosmids

### 3-Hydroxypropionate Cycle Components Identified in C. symbiosum

The 3-hydroxypropionate cycle was first identified in the phototrophic green nonsulfur bacterium Chloroflexus aurantiacus [ 37, 43] and more recently recognized in several thermophilic crenarchaeotes within the *Sulfolobales* [ 17, 18, 20, 44]. This pathway employs several enzymes typically associated with bacterial fatty acid biosynthesis, including the biotin-dependent enzyme acetyl-CoA/propionyl-CoA carboxylase [ 45]. Because archaeal lipids are typically devoid of fatty acids, the presence of acetyl-CoA/propionyl-CoA carboxylase in *Crenarchaeota* is necessary but not sufficient evidence for autotrophic carbon assimilation by the 3-hydroxypropionate cycle.

**Figure 1:**
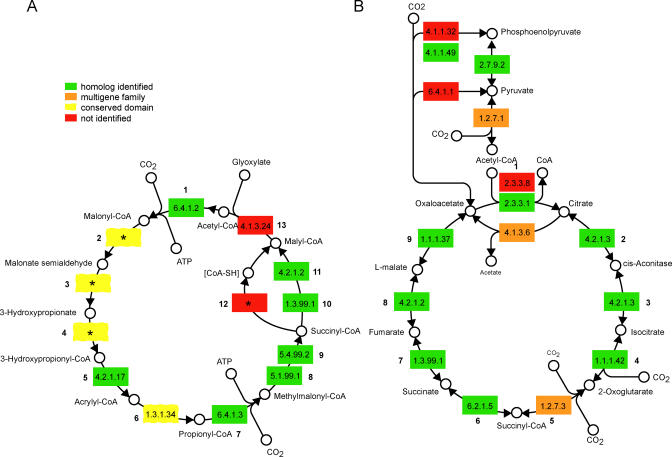
Autotrophic Carbon Assimilation Pathway Components Identified in C. symbiosum (A) 3-Hydroxypropionate Cycle Each step is mediated by the following enzymes: (1) acetyl-CoA carboxylase, (2–3) malonyl-CoA reductase, (4–6) propionyl-CoA synthase, (7) propionyl-CoA carboxylase, (8) methylmalonyl-CoA epimerase, (9) methylmalonyl-CoA mutase, (10) succinate dehydrogenase, (11) fumarase, (12) succinyl-CoA/malate CoA transferase, and (13) malyl-CoA lyase (B) TCA cycle. Each step is mediated by the following enzymes: (1) citrate synthase, (2–3) aconitase, (4) isocitrate dehydrogenase, (5) 2-oxoacid ferredoxin oxidoreductase or 2-oxoglutarate dehydrogenase, (6) succinyl-CoA synthase, (7) succinate dehydrogenase, (8) fumarase, and (9) malate dehydrogenase. In the reductive direction, the steps are reversed and citrate synthase is replaced by citrate lyase in (1). Diagrams are based on KEGG pathway maps and include enzyme classification numbers (EC) for each step in boxes when available. Box color indicates the identification status of genes encoding a particular step. See Tables 1 and 2 for more information.

Genes predicted to encode components of eight steps mediating the 3-hydroxypropionate cycle, including acetyl-CoA/propionyl-CoA carboxylase, were unambiguously identified in the C. symbiosum fosmid sequences ( Tables 1 and S1 and Figure 1). Fosmids harboring gene sequences predicted to encode acetyl-CoA/propionyl-CoA carboxylase subunits could be assembled into a single contig approximately 1.29 million bp in length containing the *C. symbiosum* SSU-LSU ribosomal RNA operon, further reinforcing this identification scheme (Hallam et al., unpublished data). Four additional steps represented by malonyl-CoA reductase [ 41] and propionyl-CoA synthase [ 42] could not be definitively identified, in part because the specific enzymes mediating these steps remain uncharacterized throughout the archaeal domain, including those archaeal groups known to express a fully catalytic form of the 3-hydroxypropionate cycle [ 18, 20]. Despite this challenge, candidates for both enzymes could be tentatively assigned based on the identification of open reading frames containing conserved domains with putative pathway-related functions. In addition to core components of the 3-hydroxypropionate cycle, a biotin ligase *(birA)* required for assembly and activation of the carboxylase complex in vivo was also identified ( Tables 1 and S1). Consistent with biochemical observations in M. sedula [ 18, 20], homologous genes encoding succinyl-CoA/malate transferase or L-malyl-CoA lyase, required for the regeneration of acetyl-CoA from glyoxylate, were not identified in the C. symbiosum fosmid sequences.

In *C. aurantiacus,* malonyl-CoA reductase activity is associated with a bifunctional enzyme containing alcohol dehydrogenase and aldehyde dehydrogenase domains, mediating the conversion of malonyl-CoA to 3-hydroxypropionate via malonate semialdehyde [ 41]. In the C. symbiosum fosmid sequences, 14 genes predicted to encode short-chain alcohol dehydrogenase domains with the potential to mediate conversion of malonyl-CoA to malonate semialdehyde were identified ( Tables 1 and S1). One candidate in particular, identified on three fosmids (101G10, C03A05, and C04H09), was found to contain an N-terminal alcohol dehydrogenase domain 32% identical and 46% similar to the corresponding interval of malonyl-CoA reductase from C. aurantiacus. Adjacent to this domain, a 142–amino acid interval, 27% identical and 40% similar to an aldehyde dehydrogenase from *Mus musculus,* was also identified as potentially involved in the conversion of malonate semialdehyde to 3-hydroxypropionate.

In *C. aurantiacus,* propionyl-CoA synthase activity is associated with a trifunctional enzyme containing CoA ligase, enoyl-CoA hydratase, and enoyl-CoA reductase domains [ 42]. Three copies of a gene predicted to encode enoyl-CoA hydratase, one of three enzymatic steps associated with propionyl-CoA synthase activity, were identified in the C. symbiosum fosmid sequences ( Tables 1 and S1). One copy of enoyl-CoA hydratase, identified on fosmid C13E07 contained a 182–amino acid interval 27% identical and 40% similar to propionyl-CoA synthase from C. aurantiacus. Immediately upstream of this open reading frame, a second gene predicted to encode a CoA binding protein related to acyl-CoA synthase, a potential effector of the indeterminate CoA ligase step, was also identified. In C. aurantiacus the enoyl-CoA reductase domain of propionyl-CoA synthase belongs to the family of zinc-binding dehydrogenases that includes NAD(P)H-dependent crotonyl-CoA reductases [ 42]. Seven copies of genes predicted to encode zinc-binding alcohol dehydrogenases with potential to mediate propionyl-CoA synthase activity were identified in the C. symbiosum fosmid sequences ( Tables 1 and S1). One candidate in particular, identified on three fosmids (C04F04, C05C02, and C13G08) was 29% identical and 48% similar to NAD(P)H-dependent crotonyl-CoA reductase from *Silicibacter sp. TM1040*. In all three instances, this gene was found in an operon with a second gene predicted to encode the beta subunit of citrate lyase (see below).

Taken together, these observations suggest that C. symbiosum has the genetic potential to encode a modified version of the 3-hydroxypropionate cycle for CO _2_ incorporation into biomass. Biochemical and physiological studies remain necessary, however, to fully validate this hypothesis and determine the specific pathway of acetyl-CoA regeneration in the absence of a glyoxylate shunt.

### TCA Cycle Components Identified in C. symbiosum

The steps of the TCA cycle define a central clearinghouse in cellular metabolism, linking the oxidative breakdown of sugars, fats, and proteins with the production of precursor molecules for biosynthesis and energy metabolism. Alternatively, the TCA cycle can be reversed in certain prokaryotic organisms, resulting in the formation of oxaloacetate from two molecules of CO _2_, thereby providing an alternative route for autotrophic carbon assimilation. The enzyme citrate synthase is responsible for the conversion of oxaloacetate and acetyl-CoA to citrate in the oxidative branch of the TCA cycle. The TCA cycle operating in the reductive direction requires the activity of ATP citrate lyase (also known as ATP citrate synthase) first described in Chlorobium limicola [ 46, 47]. ATP citrate lyase is encoded by the genes *aclA* and *aclB,* which when expressed form a heteromeric enzyme complex capable of cleaving citrate to produce oxaloacetate and acetyl-CoA [ 48]. An evolutionarily related alternative to ATP citrate lyase has recently been described in the hydrogen-oxidizing thermophilic bacterium *Hydrogenobacter thermophilus,* mediating citrate cleavage in conjunction with the enzymes citryl-CoA synthase *(ccs)* and citryl-CoA lyase *(ccl)* [ 49, 50].

Genes with the potential to encode components of nine steps mediating the oxidative or reductive TCA cycles were identified in the C. symbiosum fosmid sequences ( Tables 2 and S1 and Figure 1B). In addition to core TCA components, genes encoding phosphoenolpyruvate carboxykinase and phosphoenolpyruvate synthase were identified, consistent with an anapleurotic role for these enzymes in balancing carbon transfer into and out of TCA-dependent pathways ( Tables 2 and S1 and Figure 1B). No homologue for pyruvate carboxylase mediating the ATP-dependent carboxylation of pyruvate to oxaloacetate was recovered from the C. symbiosum fosmid sequences. A relatively small subset of TCA components was not identified or could not be readily assigned due to high levels of similarity between close relatives with varying substrate specificities. These include subunits of citrate lyase and ferredoxin oxidoreductase described below.

In the case of the oxidative TCA cycle, a near-complete pathway for the conversion of citrate to oxaloacetate could be reconstructed based on the identification of at least one copy of genes predicted to encode citrate synthase, aconitase, isocitrate dehydrogenase, succinyl-CoA synthase, succinate dehydrogenase, fumarase, and malate dehydrogenase ( Tables 2 and S1 and Figure 1B). Step 5 (see Figure 1B) mediating the conversion of 2-oxoglutarate to succinyl-CoA could not be unequivocally determined because of the high degree of amino acid conservation between the group of ferredoxin oxidoreductases responsible for synthesis or cleavage of pyruvate, 2-isoketovalerate, or 2-oxoglutarate ( Tables 2 and S1). Although four fosmids containing operons predicted to encode 2-oxoacid:ferredoxin oxidoreductase subunits were identified in the C. symbiosum sequences (C01A08, C17D04, C05B02, and C05G02), close examination of overlapping and adjacent intervals found them to be syntenic, consistent with common coverage of an equivalent genomic interval derived from separate donors ( Table S1, unpublished data). In *Clostridium thermoaceticum,* pyruvate:ferredoxin oxidoreductase has been shown to function in both oxidative decarboxylation of pyruvate to CO _2_ and acetyl-CoA, and the carboxylation of acetyl-CoA to form pyruvate [ 51]. It is possible that *C. symbiosum,* like *C. thermoaceticum,* encodes a multifunctional enzyme complex mediating forward and reverse reactions in one or more of the TCA-dependent steps. Functional studies remain necessary, however, to determine the specificity of this complex towards pyruvate, 2-isoketovalerate, or 2-oxoglutarate.

In the case of the reductive TCA cycle, no homologues for *aclA, aclB, ccs,* or *ccl* were identified in the C. symbiosum fosmid sequences. However, three fosmids (C04F04, C05C02, and C13G08) containing genes predicted to encode the beta subunit of citrate lyase *(citE),* mediating the conversion of citryl-CoA to acetyl-CoA and oxaloacetate were identified ( Tables 2 and S1). In bacteria, *citE* exists as part of an operon containing genes encoding citrate-CoA transferase *(citF),* and an acyl carrier protein subunit *(citD).* Bacterial homologues of citrate lyase play defined roles in citrate fermentation pathways [ 52, 53]. However, little is known about the functional aspects of archaeal citrate lyases. A similar case to C. symbiosum has been described in the facultative heterotrophic crenarchaeon, *Thermoproteus tenax,* where a single gene predicted to encode the beta subunit of citrate lyase *(citE)* was identified in the draft genome sequence [ 54]. No direct homologues for *citD* or *citF* were identified. However, the authors speculate that two genes adjacent to *citE,* predicted to encode acetyl-CoA synthetase and acety-CoA transferase/carnitine dehydratase have the potential to fill in for the missing citrate-CoA transferase and acyl carrier subunits, respectively [ 54]. At present, ATP citrate lyase activity in T. tenax remains unmeasured, although ^13^C-labeling studies in a close relative, Thermoproteus neutrophilus, successfully measured incorporation patterns consistent with the operation of a reductive TCA cycle in autotrophically grown cells [ 22, 24]. Similar to *T. tenax,* no direct homologues of *citF* or *citD* were identified in the C. symbiosum fosmid sequences, although unlinked genes predicted to encode acetyl-CoA synthetases were identified on four separate fosmids (C01C01, C17E03, C07D05, and C13E07). In contrast to *T. tenax,* no homologues for acety-CoA transferase/carnitine dehydratase were identified.

Taken together, the data suggests that C. symbiosum utilizes either the oxidative TCA cycle in the consumption of organic carbon and in the production of intermediates for amino acid and cofactor biosynthesis, or a horseshoe version of the TCA cycle charting an oxidative branch between citrate and 2-oxoglutarate and a reductive branch between oxaloacetate and succinate for biosynthetic purposes alone.

### A Potential Mode of Ammonia Oxidation in C. symbiosum

**Figure 2:**
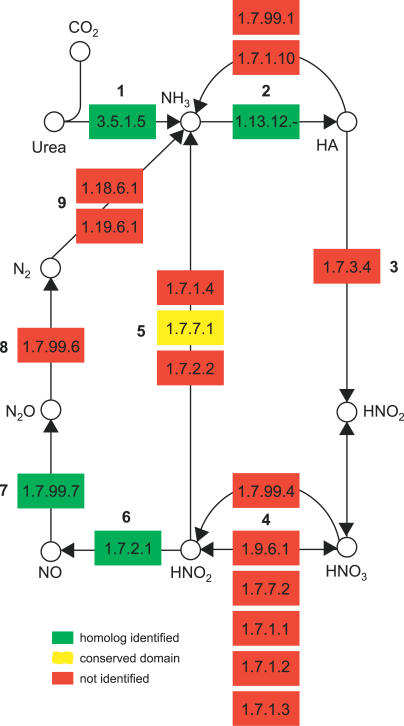
Ammonia Oxidation Pathway Components Identified in C. symbiosum Each step is mediated by the following enzymes: (1) urease, (2) ammonia monooxygenase, (3) hydroxylamine oxidoreductase, (4) nitrate reductase, (5) ferredoxin-nitrite reductase or formate-dependent nitrite reductase, (6) nitrite reductase, (7) nitric-oxide reductase, (8) nitrous-oxide reductase, and (9) nitrogenase. Box color indicates the identification status of genes encoding a particular step. NH _3_, ammonia; HA, hydroxylamine; HNO _3_, nitrate; HNO _2_, nitrite; NO, nitric oxide; N _2_O, nitrous oxide; N _2_, dinitrogen. Diagrams are based on KEGG pathway maps and include enzyme classification numbers (EC) for each step in boxes when available. See Table 3 for more information.

Chemolithoautotrophic ammonia oxidation produces energy and reducing equivalents used for cell growth, carbon assimilation, and the generation of a proton motive force. In bacteria such as Nitrosomonas europaea and *Nitrosospira multiformis,* ammonia monooxygenase composed of α, β, and γ membrane-bound subunits, encoded by the genes *amoA, amoB,* and *amoC,* respectively, catalyzes the conversion of ammonia (NH _3_) to hydroxylamine (NH _2_OH). In these, NH _2_OH is subsequently converted to nitrite through the activity of hydroxylamine oxidoreductase, a phylogenetically unique homotetramer containing eight heme groups [ 55]. Among ammonia-oxidizing bacteria, up to three copies of the *amo* operon may be present, with conserved gene order, *amoCAB* [ 55]. Two fosmids (C07D08 and C18D02) containing genes encoding putative α, β, and γ subunits, co-located over an approximately 6-Kb interval were identified in the C. symbiosum fosmid sequences ( Tables 3 and S1 and Figures 2, 4, and S1). The *C. symbiosum amoA, amoB,* and *amoC* genes were predicted to encode proteins 26% identical and 40% similar, 25% identical and 44% similar, and 50% similar and 32% identical to the corresponding α, β and γ subunits from *N. multiformis, N. oceani,* and *N. europaea,* respectively. A second unlinked gene encoding a γ subunit, 31% identical and 51% similar to the γ3 subunit from N. europaea was also identified in the C. symbiosum fosmid sequences ( Tables 1 and S1 and Figure 4B). Analysis of predicted transmembrane domains affiliated with C. symbiosum ammonia monooxygenase subunits identified 6, 2, and 4 membrane spanning intervals in the α, β, and γ subunits respectively, compared with 6, 2, and 6 membrane spanning intervals for related subunits in N. europaea (see Materials and Methods).

**Table 3:**
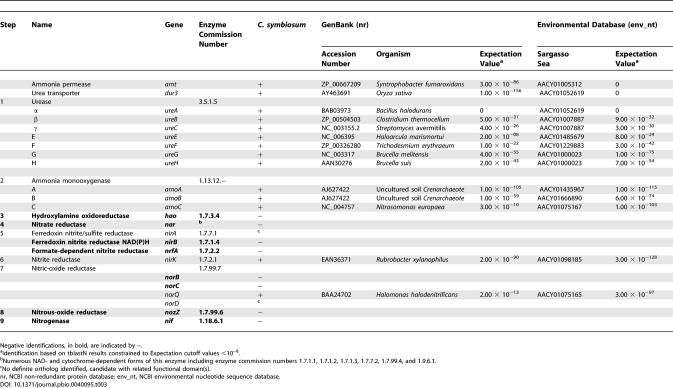
Nitrogen Cycle Components Identified on C. symbiosum Fosmids

Bacterial ammonia monooxygenase subunits share close structural and functional similarity with particulate methane monooxygenase subunits [ 56, 57]. Given this relationship, a search was conducted for downstream effectors of aerobic methane oxidation in the C. symbiosum sequence. Two steps in the tetrahydrofolate-dependent (H4F) pathway of methane oxidation encoded by a bifunctional 5,10-methylene-H4F dehydrogenase/methenyl-H4F cyclohydrolase were identified [ 58]. However, neither formyl-H4F synthase nor formate dehydrogenase homologues could be found, suggesting a role for the bifunctional enzyme in folate biosynthesis rather than methane oxidation. Moreover, no gene identifications could be made for components of the tetrahydromethanopterin-dependent (H4MPT) pathway, including methylene-H4MPT dehydrogenase, methenyl-H4MPT cyclohydrolase, formylmethanofuran-H4MPT dehydrogenase, or formylmethanofuran dehydrogenase [ 58].

Although homologues for all three *amo* subunits were identified in the C. symbiosum fosmid sequences, downstream effectors of the canonical bacterial pathway including hydroxylamine oxidoreductase and two cytochromes, *c*_554_ and *cm*_552_ *,* required for electron flow to ubiquinone [ 55] were not be identified. The identification of *amo* gene sequences and the absence of *hao, c*_554_ *,* and *cm*_552_ homologues, suggests that nonthermophilic *Crenarchaeota* may use alternative mechanisms for channeling electrons derived from ammonia oxidation into the electron transport system. Consistent with this hypothesis, 34 genes predicted to contain blue type (I) copper binding domains (similar to the plastocyanin/azurin family) with the potential to substitute for cytochromes in archaeal energy metabolism [ 59] were identified in the C. symbiosum fosmid sequences ( Table S1). Several of these genes were found to exist in operons containing up to four closely related copies (C01B01, C02G05, C05C02, and C08B04) or in combination with genes encoding Rieske iron-sulfur cluster proteins and subunits related to the cytochrome b6 *f* complex (C01C01, C03H05, C17E03, and C13A11) [ 60, 61].

Two transporters potentially involved in the delivery of reduced nitrogen compounds were identified in the C. symbiosum fosmid sequences ( Tables 3 and S1). In the first case, five copies of a gene 47% identical and 62% similar to a predicted ammonia permease *(amt)* from the anaerobic propionate oxidizing bacterium Syntrophobacter fumaroxidans were identified ( Tables 3 and S1). In the second case, two copies of a gene predicted to encode a urea transporter, 52% identical and 70% similar to an ATP-dependent urea transporter *(dur3)* from *Oryza sativa,* was identified ( Tables 3 and S1 and Figure 2). Immediately adjacent to the predicted *dur3* homologue, an operon encoding three core urease *(ure)* subunits arranged in the order *ureCBA* was identified, followed by a second operon encoding five additional urease accessory genes arranged in the order *ureDEFGH* in opposite orientation to the *dur3/ureCBA* operon ( Tables 3 and S1). The most conserved homologues from known organisms for all seven of the *ure* subunits were derived from bacterial groups ( Table 3). Several ammonia oxidizing bacteria, including Nitrosospira sp. strain NpAV and N. oceani encode functional urease operons with related subunit composition to the urease observed in the C. symbiosum fosmid sequences [ 62]. In *Nitrosospira,* urease provides a complementary source of ammonia and carbon dioxide for chemolithoautotrophic growth in low pH environments and a mechanism for alkalization of the surrounding microenvironment [ 62, 63].

In addition to components of a potential ammonia oxidation pathway, four fosmids (C01A05, C02B01, C01D10, and C15E09) containing genes predicted to encode multicopper oxidases related to nitrite reductase *(nirK)* ( Tables 3 and S1 and Figures 2 and S2) and three fosmids (C07C04, C20E06, and C08B03) containing genes predicted to encode nitric oxide reductase subunits *(norQ* and *norD)* were identified in the C. symbiosum fosmid sequences ( Tables 3 and S1 and Figure 2). The predicted nitrite reductase was 54% identical and 67% similar to a copper-containing nitrite reductase from the actinomycete Frankia sp. CcI3, a nitrogen-fixing symbiont of nonleguminous plants ( Tables 3 and S1). The nitric oxide reductase homologue was found to exist in an operon with a second gene predicted to contain a 136 amino acid interval 25% identical and 44% similar to the nitric oxide reductase activation protein from *Thiobacillus denitrificans,* and a third gene 31% identical and 46% similar to a conserved archaeal protein from Sulfolobus acidocaldarius related to electron-transferring flavoproteins. In addition to the predicted nitrite reductase and nitric oxide reductase, four fosmids (C06D08, C09D08, C12D02, and C13F02) predicted to encode ferredoxin sulfite/nitrite reductase subunits, 40% identical and 57% similar to ferredoxin sulfite/nitrite reductase beta subunits from *Thermus thermophilus,* were identified. In all instances, the two beta subunits formed part of a larger gene cluster encoding a rhodanese-related sulfurtransferase, thiazole biosynthesis protein, and thioredoxin reductase, suggesting a role in assimilatory sulfur metabolism.

As downstream components of a potential ammonia oxidation pathway, copper containing nitrite reductase and nitric oxide reductase could play a role in determining tolerance to nitrite and nitric oxides, respectively, or under limiting oxygen concentrations, allow for the use of nitrous oxide as an alternative electron acceptor [ 64] *.* A similar set of observations has been experimentally verified in *N. europaea,* where a periplasmic copper-type nitrite reductase and a nitric oxide reductase complex were found to protect cells from the toxic effects of nitrite and nitric oxide, respectively [ 65, 66].

Taken together, the data are consistent with the hypothesis that C. symbiosum is capable of utilizing reduced nitrogen compounds, including urea and ammonia, to generate cellular energy. Moreover, the identification of genes involved in the metabolism of nitrogen oxides provides a plausible mechanism for removing the toxic byproducts of ammonia oxidation. Functional studies remain necessary to validate these hypothetical pathways, and to determine the specific pathway of ammonia conversion in the absence of a defined homologue for hydroxylamine oxidoreductase.

### Carbon Assimilation and Ammonia Oxidation Pathway Ggene Sequences in Planktonic *Crenarchaeota*

#### Autotrophic carbon assimilation pathway genes

To identify planktonic crenarchaeal homologues of C. symbiosum genes potentially involved in the 3-hydroxypropionate and TCA cycles, searches were conducted with the set of identified C. symbiosum open reading frames, queried against GenBank and the SAR environmental database [ 26] ( Tables 1, 2, S2, and S3). In the case of defined C. symbiosum TCA cycle components, the top-scoring GenBank IDs were distributed evenly among crenarchaeal (31%), euryarchaeal (31%), and bacterial (38%) groups. In contrast, the overwhelming majority (83%) of defined 3-hydroxypropionate cycle components identified in this study were distributed among crenarchaeal (43%) and euryarchaeal (50%) groups. In two instances, the top-scoring GenBank subjects came from fosmid clones derived from uncultivated groups representing planktonic *Crenarchaeota* ( Tables 1 and 2). In all cases, the highest-scoring BLAST identities for 3-hydroxypropionate and TCA cycle components were identified in the environmental database composed of gene sequences recovered from the SAR ( Tables 1 and 2), consistent with the presence of genotypes representing planktonic *Crenarchaeota* across the combined set of seven sequenced sample bins [ 26].

**Figure 3:**
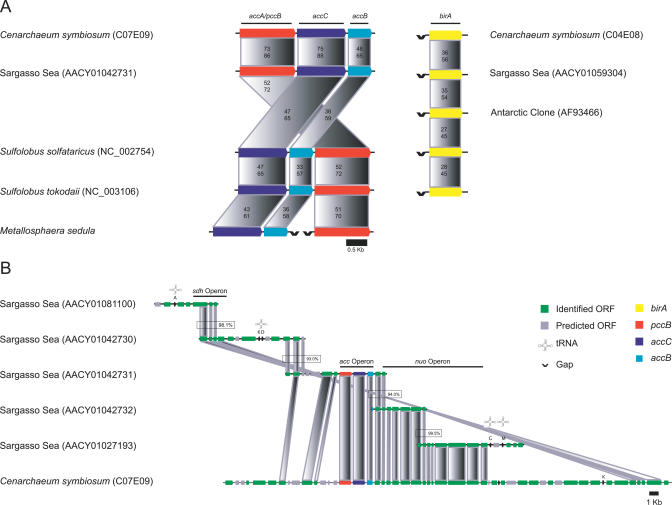
Relationship of C. symbiosum Acetyl-CoA Carboxylase *(acc)* Operon to Other Crenarchaeal Lineages (A) Protein identity (top) and similarity (bottom) percentages between predicted gene products identified in *C. symbiosum,* a contig identified in the environmental database of SAR WGS sequences, and a subset of thermophilic *Crenarchaeota* are shown within the corresponding shaded boxes. (B) Comparison of the genomic interval containing the *C. symbiosum acc* operon with several contigs identified in the environmental database of SAR WGS sequences. Genes shared in common between genomic intervals are connected by shaded boxes. Values in clear boxes represent the nucleotide identity between overlapping SAR contigs.

In members of the *Sulfolobales,* genes encoding acetyl-CoA carboxylase subunits are typically arranged in a single operon in the order *accCBA* [ 20, 21]. In *C. symbiosum,* genes encoding acetyl-CoA carboxylase subunits were arranged in a single operon with the order *accACB*. A contig assembled from WGS sequence recovered from the SAR (AACY01042731) contained an intact *acc* operon with subunit organization identical to that of C. symbiosum ( Figure 3A). A biotin ligase *(birA)* containing contig identified in the SAR dataset (AACY01093101) exhibited a similarly close relationship to the *birA* interval identified in C. symbiosum fosmid sequences ( Figure 3A, Table S2, and unpublished data).

**Figure 4:**
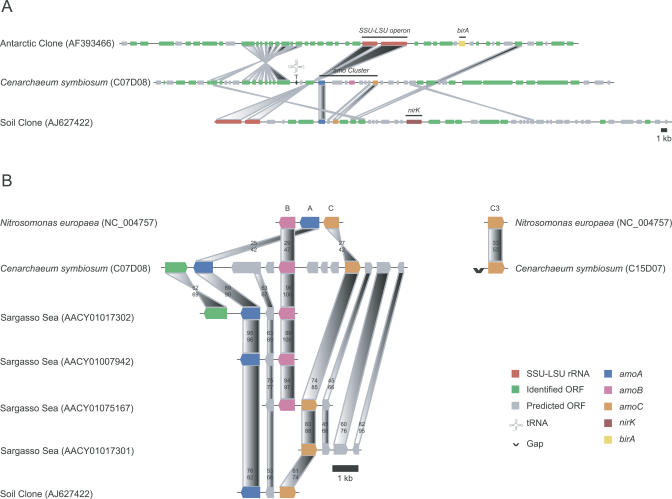
Relationship of C. symbiosum Ammonia Monooxygenase *(amo)* Gene Cluster to Other Uncultivated Crenarchaeal Sequences (A) Comparison of the genomic interval containing ammonia monooxygenase genes in C. symbiosum with two related environmental fosmid sequences, one obtained from mesopelagic seawater (AF393466) and the other from terrestrial soil (AJ627422). (B) Comparison of the order and composition of the C. symbiosum ammonia monooxygenase *(acc)* gene cluster and unlinked *amoC3* locus in relation to the bacterial operon from *N. europaea,* several highly related contig intervals identified in the environmental database of SAR WGS sequences, and a terrestrial soil sequence. Protein identity (top) and similarity (bottom) percentages between predicted gene products identified in C. symbiosum are shown within the corresponding shaded boxes. See Tables S1– S3 for more information.

Comparison of deduced amino acid sequences revealed that subunits of the *acc* operon identified on AACY01042731 were more closely related to C. symbiosum than to other archaeal groups ( Figure 3B and unpublished data) *.* Moreover, predicted gene identity over the length of AACY01042731 closely matched that of C. symbiosum in regions flanking the *acc* operon ( Figure 3B). Using this contig as a starting point for nucleotide BLAST surveys of the environmental database, additional overlapping contigs (AACY01081100, AACY01042730, AACY01042732, and AACY01027193) were identified in the environmental database ( Figure 3B and Table S2). Conceptual translation of these sequence intervals revealed a high degree of conservation between overlapping regions more distal to the *C. symbiosum acc* operon, including a large operon predicted to encode the complete set of NADH-ubiquinone oxidoreductase subunits immediately downstream of the *acc* operon, and a second operon containing succinate dehydrogenase subunits situated several kilobases upstream ( Figure 3B). Paired-end analysis of individual reads from the SAR trace files confirmed the order and orientation of the set of contigs identified in the environmental database based on homology to the *C. symbiosum acc* interval (unpublished data). SAR contigs with the potential to encode the complete set of 3-hydroxypropionate components identified in the C. symbiosum fosmid sequences were identified in the environmental database. In each instance at least one contig greater than 3 Kb was identified sharing significant conservation of gene content and order in regions adjacent to the original query sequence ( Table S2).

#### Ammonia assimilation and oxidation genes

To identify homologues of the ammonia monooxygenase subunits predicted in the C. symbiosum fosmid sequences, targeted searches were conducted against GenBank and the environmental database of SAR whole-genome shotgun sequences ( Table 3). Top scoring GenBank subjects for α and β subunits were associated with an uncultured soil crenarchaeote (AJ627422) [ 28]. The γ subunit was most similar to the γ3 subunit from N. europaea ( Table 3). In all cases, the highest scoring BLAST identities for ammonia monooxygenase subunits were identified in the environmental database composed of gene sequences recovered from the SAR ( Tables 3 and S3).

Comparison of the genetic neighborhood surrounding the *amo* gene cluster in C. symbiosum with the terrestrial soil clone (AJ627422) revealed little conservation of genes adjacent to the *amoA* and *amoB* loci ( Figure 4A). In contrast, a planktonic crenarchaeal fosmid clone (AF393466) recovered from Antarctic waters contained a block of nine predicted open reading frames completely syntenic at the amino acid level to a 6-Kb genomic interval upstream of the *amoA* gene identified in C. symbiosum ( Figure 4A) [ 33]. Similarly, three contigs assembled from WGS sequence recovered from the SAR (AACY01017302, AACY01007942, and AACY01075167) contained homologous genes encoding α and γ, and γ and β subunits, respectively ( Figure 4B and Table S3). A fourth contig (AACY01017301) containing the *amoB* gene was found to contain several adjacent open reading frames predicted to encode hypothetical proteins shared in common with the *amo* gene cluster in C. symbiosum ( Figure 4B). Despite a reduction in the distance between genes encoding ammonia monooxygenase subunits, SAR contigs shared additional gene content with C. symbiosum in regions nested within and flanking the *amo* interval ( Figure 4B). Predicted genes within these intervals, although hypothetical, are conserved and therefore of potential functional interest in the context of ammonia oxidation. In addition to ammonia monooxygenase subunits, 47 sequences containing at least one copy of a gene predicted to contain blue type (I) copper binding domains (related to the plastocyanin/azurin family) were identified in the SAR with homology to C. symbiosum ( Table S3), and one copy was identified on an environmental crenarchaeal fosmid recovered from mesopelagic seawater (AY316120).

**Figure 5:**
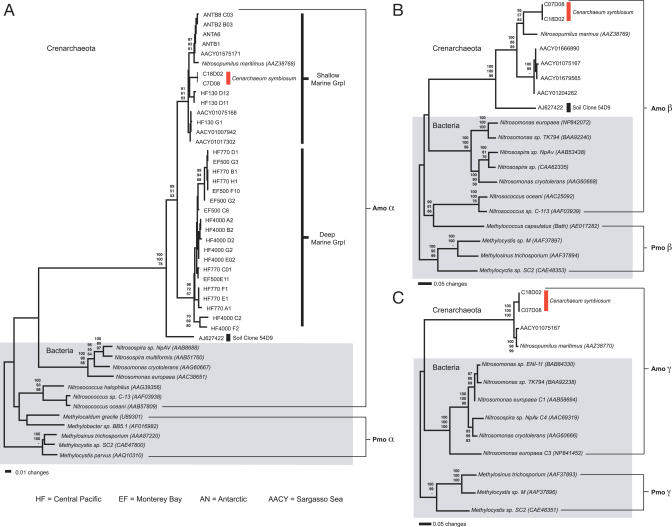
Phylogenetic Comparison of Ammonia Monooxygenase α, β, and γ Subunits Identified in C. symbiosum and Environmental DNA Libraries (A) α, (B) β, and (C) γ. The taxonomic distribution of α subunits includes sequences recovered from environmental library screening with degenerate primers targeting the *amoA* locus (see Materials and Methods). Distance (top), parsimony (middle), and maximum-likelihood (bottom) bootstrap values providing more than 50% support are indicated. Distance and parsimony values are based on 1,000 replicates each, and maximum-likelihood values are based on 10,000 replicates. Trees are rooted with the corresponding subunits for particulate methane monooxygenase.

To further explore the potential link between energy metabolism in C. symbiosum and planktonic *Crenarchaeota,* degenerate *amoA* PCR primers based on the C. symbiosum sequence were designed and used to screen bacterial artificial chromosomes (BAC) and fosmid libraries of planktonic microbes from the Central Pacific, Monterey Bay, and the Antarctic peninsula (see Materials and Methods) [ 33, 67, 68]. A total of 26 *amoA* sequences were recovered from the combined library set. Phylogenetic comparison of deduced amino acid sequences for the α subunits revealed distant but significant similarities to homologous genes in bacteria ( Figure 5A, S1A). Genes encoding α subunits recovered from environmental libraries were partitioned between distinct shallow (0–130 m) and deep marine clades (500–4,000 m), with C. symbiosum and SAR sequences falling within the shallow marine clade ( Figure 5A). Overall, a subset of environmental sequences recovered from the water column at 130 m in the Central Pacific was most closely related to the predicted C. symbiosum α subunit *.* Similar tree topologies were observed for β and γ subunits based on comparison of deduced amino acid sequences for *amoB* and *amoC* genes identified in GenBank and the SAR datasets ( Figures 5B, 5C, S1B, and S1C).

The most conserved homologues for *C. symbiosum–*associated ammonia permease, urea transporter, and urease subunits were all found in the environmental database of gene sequences from the SAR ( Table 3). A contig assembled from WGS sequence recovered from the SAR (AACY01052619) contained homologous genes encoding the urea transporter, the core urease operon *(ureABC),* and two additional accessory genes *(ureEF).* ( Table S3 and unpublished data). An overlapping contig (AACY01000023) containing the remaining urease accessory genes *(ureEFGH)* was also identified. The presence of ammonia permease, linked urea transport, and catabolism genes in *C. symbiosum,* as well as in sequences derived from the SAR, suggest the capacity for generation and utilization of reduced nitrogen compounds for either amino acid biosynthesis, energy production, or both by planktonic *Crenarchaeota*. SAR contigs encoding the complete set of ammonia monooxygenase components identified in the C. symbiosum fosmid sequences were identified in the environmental database. In each instance, at least one contig greater than 2.5 Kb shared significant conservation of gene content and order in regions adjacent to the C. symbiosum sequence ( Table S3).

#### Sequence coverage and synteny between C. symbiosum genes and environmental databases

To explore the distribution and coverage of crenarchaeal sequences in the SAR, contigs containing *acc, birA, amo,* and *ure* genes homologous to C. symbiosum were used to identify individual WGS reads from each of seven separate SAR shotgun DNA libraries (see Materials and Methods) [ 26]. The distribution of sequences encoding crenarchaeal homologues for this gene set varied considerably between samples, with SAR samples 2, 3, and 4 containing the majority of sequences, while SAR samples 5, 6, and 7 contained none ( Figure S3). Although it was twice as twice as large as SAR samples 2, 3, and 4, and collected in the same water on the same day as SAR sample 2, SAR sample 1 contained far fewer WGS reads encoding crenarchaeal homologues for *acc, birA, amo,* and *ure* genes. This observation is presumably the result of contamination in SAR sample 1 that masked the representation of endogenous genotypes. Similar results for each sample bin were obtained in searches for crenarchaeal SSU ( Figure S3) and LSU (unpublished data) sequences (see Materials and Methods). These observations are consistent with sampling parameters, including the pore size of prefiltration and collection filters used (0.8–0.1 μm for SAR sample 1 and 7, 0.8–0.22 μm for SAR samples 24, 20–3.0 μm for SAR sample 5, and 3–0.8 μm for SAR sample 6), and collection dates (2/25/2003 for SAR samples 3–4, 2/26/2003, for SAR samples 1–2, and 5/15/2003 for SAR samples 5–7). The late February collection dates for SAR samples 1–4 were at a time of deep water mixing [ 26]. On average five homologous genes were identified for *acc, birA, amo, dur3,* and *ure* genes in SAR samples 2–4, consistent with low to moderate density representation of planktonic crenarchaeal genotypes in these samples. Moreover, the absence of a defined crenarchaeal signal for SAR sample 7, targeting the same size fraction as SAR samples 2–4, provides evidence for a change in the surface abundance of planktonic *Crenarchaeota* in the SAR over the 3-mo interval between sample collection. Sequence representation and paired-end information, combined with the identification and assembly of extended genomic intervals, provides a starting point for phylogenetic binning of presumptive planktonic crearchaeal clones ( Tables S2 and S3). A first and essential step in conducting comparative genomic studies using unassembled environmental sequence data.

## Discussion

*Crenarchaeota* although ubiquitous and abundant in the marine environment have remained metabolically uncharacterized, in large part due to their resistance to cultivation. The recent cultivation of a planktonic crenarchaeal isolate represents a breakthrough in this regard [ 29] and should provide an invaluable biochemical system in which to test and extend the hypotheses put forward in environmental genomic studies. Over one decade ago cultivation-independent analyses of environmental samples led to the discovery of planktonic *Crenarchaeota* [ 1, 3]. Application of these techniques continues to provide important information on the nature, distribution, and comparative genomics of marine *Crenarchaeota*. At present the molecular mechanisms underlying observed patterns and processes of autotrophic carbon assimilation and ammonia oxidation in marine *Crenarchaeota* remain unresolved. The identification of candidate genes involved in these pathways is an initial step in understanding the physiology and ecology of marine *Crenarchaeota* and identifying targets and building hypotheses for functional testing.

Multiple components of the 3-hydroxypropionate cycle of autotrophic carbon assimilation were identified in the C. symbiosum fosmids and in databases containing environmental sequences representing uncultivated planktonic *Crenarchaeota*. In every instance, the local genetic neighborhood surrounding the gene of interest was related between the various marine crenarchaeal lineages. Several components of the cycle could not be unequivocally determined on the basis of homology, but could be tentatively identified by of domain relatedness to corresponding pathway functions in the bacterial domain. For instance, candidate genes with the potential to encode components of malonyl-CoA reductase and propionyl-CoA synthase were identified with homologous regions to known genes encoding these activities in C. aurantiacus. Several steps in the pathway, however, remain unknown, including the mode of acetyl-CoA regeneration in the absence of genes typically involved in glyoxylate metabolism. Taken together, the data are consistent with functional studies in more distantly related thermophilic *Crenarchaeota,* including T. neutrophilus [ 22, 23] and multiple members of the *Sulfolobales* [ 17, 18, 20, 21]. Based on this previous work and the analyses reported here, C. symbiosum and its planktonic relatives have the potential to employ a modified version of the 3-hydroxypropionate cycle for autotrophic carbon assimilation.

The pathway of crenarchaeal ammonia oxidation appears to be different from well-defined bacterial systems, given the apparent absence of canonical downstream components typically associated with electron flow between hydroxylamine and the ubiquinone pool. However, a good case for the process of ammonia oxidation can be made given the primary and secondary homology between marine crenarchaeal and bacterial ammonia monooxygenase subunits. This role is further supported by: the presence of genes encoding small copper proteins with the potential to substitute for cytochromes in mediating electron flow [ 60, 61], transport systems for reduced nitrogen compounds including ammonia and urea, the presence of downstream genes encoding enzymes potentially involved in nitrite and nitric oxide detoxification, the absence of downstream genes involved in aerobic methane oxidation, and the physical and chemical oceanographic data linking crenarchaeal abundance with byproducts of ammonia oxidation [ 4, 14– 16, 25]. The identification of crenarchaeal *amo* homologues in complex genomic libraries harboring uncultivated crenarchaeotes genomes and in a marine crenarchaeal isolate cultivated on bicarbonate and ammonia as sole carbon and energy sources [ 29], reinforces the hypothesis that “free-living” marine crenarchaeota employ an ammonia oxidation pathway for energy generation.

Comparison of genomic features shared between C. symbiosum and planktonic marine *Crenarchaeota* reveals several emergent themes. Although dramatically divergent at the nucleotide level (more than 15% difference in G+C content), C. symbiosum shares striking homology with planktonic *Crenarchaeota* in terms of gene identity, operon organization, and content. This kinship appears to scale across oceanic provinces based on the identification of syntenic intervals between an archaeal fosmid recovered from Antarctic waters, assembled sequences from the Sargasso Sea, and C. symbiosum genomic DNA itself. In every instance examined, C. symbiosum gene products described in this study were most closely related to homologues recovered from planktonic *Crenarchaeota*. This protein-based homology is suggestive, given the divergent selective forces experienced by symbiotic and free-living crenarchaeal lineages, of significant shared metabolic capacity. The variation in crenarchaeal ammonia monooxygenase related subunits with water depth is also of ecological interest, given the potential differences in dissolved oxygen concentrations between surface and benthic waters or between planktonic and symbiotic environments.

Environmental surveys have shown *Crenarchaeota* to be dominant members of planktonic microbial communities [ 1– 3, 69, 70]. Radiocarbon and stable carbon isotope tracer studies have implicated dissolved inorganic carbon as a carbon source for planktonic *Crenarchaeota* [ 14– 16]. We show here that genetic information derived from C. symbiosum can be used in the reconstruction of plausible biochemical mechanisms for autotrophic carbon assimilation, and a mode of energy production required to sustain it. These mechanisms likely converge on a facultative chemoautotrophic metabolism, based on the eurybathyal distribution of *Crenarchaeota* in the marine environment. The identification of gene sequences involved in 3-hydroxypropionate cycle, TCA cycle, and ammonia oxidation pathways in C. symbiosum provides a framework for inferring the genetic, biochemical, and physiological properties of marine *Crenarchaeota* and a database for designing molecular tools to test these hypotheses directly in the world's oceans.

## Materials and Methods

### Sample preparation and fosmid library construction

C. symbiosum cell enrichment and DNA extraction from sponge tissue and fosmid library construction have been previously described [ 32]. High molecular weight DNA purified from A. mexicana cell enrichments was used to construct two 32–45 Kb insert fosmid DNA libraries [ 31]. Briefly, tissue from an individual Axinella mexicana sponge was homogenized and enriched for prokaryotic cells by differential centrifugation [ 31]. High-molecular-weight DNA from this cell preparation was partially digested with *Sau*3AI (Promega, Madison, Wisconsin, United States) and treated with heat-labile phosphatase (HK phosphatase; Epicentre, Madison, Wisconsin, United States). The partially digested genomic DNA was ligated into pFOS [ 71] digested previously with *Aat*II, phosphatase treated (HK phosphatase), and subsequently digested with *Bam*HI. Ligated DNA was packaged using the Gigapack XL packaging system (Stratagene, La Jolla, California, United States) selecting for DNA inserts of 35 to 45 kilobase-pairs, and used to transfect E. coli DH10B cells (Invitrogen, Carlsbad, California, United States ). Transfected cells were selected on LB agar containing 15 μg/ml chloramphenicol (LB _cm15_). Resulting fosmid clones were picked into 96-well microtiter dishes containing LB _cm15_ with 7% glycerol and stored at −80 °C.

### Shotgun sequencing library preparation from fosmid clones

Approximately 3–5 μg of selected fosmid DNA was randomly sheared to 3–4 kb fragments (25 cycles at speed code 12) in 100-μL volume using a HydroShear (GeneMachines, San Carlos, California, United States). The sheared DNA was immediately blunt end–repaired at room temperature for 40 min using 6 U of T4 DNA Polymerase (Roche, Mountain View, California, United States), 30 U of DNA Polymerase I Klenow Fragment (New England Biolabs, Beverly, Massachusetts, United States), 10 μL of 10 mM dNTP mix (Amersham Biosciences, Piscataway, New Jersey, United States), and 13 μL of 10× Klenow Buffer in 130-μL total volume. After incubation the reaction was heat inactivated for 15 min at 70 ^°^C, cooled to 4 ^°^C for 10 min, and then frozen at −20 ^°^C for storage. The end-repaired DNA was run on a 1% TAE agarose gel for ˜ 30–40 min at 120 v. Using ethidium bromide stain and UV illumination, 3–4-kb fragments were extracted from the agarose gel and purified using QIAquick Gel Extraction Kit (Qiagen, Valencia, California, United States). Approximately 200–400 ng of purified fragment was blunt-end ligated for 40 min into the Sma I site of 100 ng of pUC 18 cloning vector (Roche) using the Fast-Link DNA Ligation Kit (Epicentre). Following standard protocols, 1 μL of ligation product was electroporated into DH10B Electromax cells (Invitrogen) using the GENE PULSER II electroporator (Bio-Rad, Hercules, California, United States). Transformed cells were transferred into 1000 μL of SOC and incubated at 37 ^°^C in a rotating wheel for 1 h. Cells (usually 20–50 μL) were spread on LB agar plates 22 × 22 cm containing 100 μg/mL of ampicillin, 120 μg/mL of IPTG, and 50 μg/mL of X-GAL. Colonies were grown for 16 h at 37 ^°^C. Individual white recombinant colonies were selected and picked into 384-well microtiter plates containing LB/glycerol (7.5%) media containing 50 μg/mL of ampicillin using the Q-Bot multitasking robot (Genetix, Dorset, United Kingdom). To test the quality of the library, 24 colonies were directly PCR amplified with pUC M13 −28 and −40 primers using standard protocols. Libraries are considered to pass PCR QC if they have >90% 3-kb inserts (for more information go to http://www.jgi.doe.gov/sequencing/protocols/index.html).

### Plasmid amplification

2-μL aliquots of saturated E. coli cultures (DH10B) containing pUC18 vector with random 3°4 kb DNA inserts grown in LB/glycerol (7.5%) media containing 50 μg/mL of ampicillin were added to 8 μL of a 10 mM Tris-HCl (pH 8.2) + 0.1 mM EDTA denaturation buffer. The mixtures were heat lysed at 95 ^°^C for 5 min, then placed at 4 ^°^C for 5 min. To these denatured products, 10 μL of an RCA reaction mixture (Templiphi DNA Sequencing Template Amplification Kit, Amersham Biosciences) were added. The amplification reactions were carried out at 30 ^°^C for 12–18 h. The amplified products were heat inactivated at 65 ^°^C for 10 min, then placed at 4 ^°^C until used as template for sequencing.

### Sequencing and assembly

Aliquots of the 20-μL amplified plasmid RCA products were sequenced with standard M13 −28 or −40 primers. The reactions contained 1 μL RCA product, 4 pmoles primer, 5 μL dH _2_O, and 4 μL DYEnamic ET terminator sequencing kit (Amersham Biosciences). Cycle-sequencing conditions were 30 rounds of 95 ^°^C–25 sec, 50 ^°^C–10 sec, 60 ^°^C–2 min, hold at 4 ^°^C. The reactions were then purified by a magnetic bead protocol (see research protocols at http://www.jgi.doe.gov) and run on a MegaBACE 4000 (Amersham Biosciences). Alternatively, 1 μL of the RCA product was sequenced with 2 pmoles of standard M13 −28 or −40 primers, 1 μL 5× buffer, 0.8 μL H _2_O, and 1 μL BigDye sequencing kit (Applied Biosystems, Foster City, Calfiornia, United States) at 1 min denaturation and 25 cycles of 95 ^°^C–30 sec, 50 ^°^C–20 sec, 60 ^°^C–4 min, and finally held at 4 °C. The reactions were then purified by a magnetic bead protocol (see research protocols at http://www.jgi.doe.gov/sequencing/protocols/index.html) and run on an ABI PRISM 3730 (Applied Biosystems) capillary DNA sequencer. Assembly of raw trace files was accomplished with PHRED/PRAP ( http://www.phrap.org/phredphrapconsed.html) software [ 72, 73].

### Environmental library screening for *amoA* genes

Environmental DNA libraries derived from a variety of marine sources including the Central Pacific, Monterey Bay [ 67, 74], and Antarctic waters [ 67, 74] were screened by PCR with the following degenerate primer pair designed to detect crenarchaeal amoA gene sequences (crenAMO_F 5′ ATGGTCTGGCTAAGACGM TGTA 3′ and crenAMO_R 5′ CCCACTTTGACCAAGCGGCCAT 3′). 50-μl amplification reaction mixtures contained 1 μl template DNA, 41.5 μl 1× buffer, 1 μl each 10 μM forward and reverse primer, 2.5 units TaqPlus Precision polymerase (Stratagene), 5 μl 10mM stock dNTP mixture. Amplifications were carried out using the following profile: 94 °C/2 min, ×30 cycles 94 °C/15 sec, 55 °C/30 sec, and 72 °C/30 sec, followed by a final extension at 72 °C/10 min. *amoA* amplicons were visualized on 1% agarose gels in 1× TBE and purified directly using Ultrafree-DA DNA extraction filters (Millipore, Billerica, Massachusetts, United States), followed by ethanol precipitation and resuspension in 5 μl of sterile filtered water. Purified products were sequenced bidirectionally with amoA_F and amoA_R primers on an ABI Prism 3100 DNA sequencer (Applied Biosystems) using Big Dye chemistry (PE Biosystems, Foster, California, United States) according to manufacturer's instructions. Sequences were edited manually from traces using Sequencher software V4.1.2 (Gene Codes, Ann Arbor, Michigan, United States). Analyses of predicted transmembrane spanning regions of deduced amino acid sequences for selected *amo* sequences were performed directly online with the TMHMM server v2.0 ( http://www.cbs.dtu.dk/services/TMHMM/) using default settings.

### DNA sequence analysis

Fosmid sequences were annotated using the FGENESB pipeline for automatic annotation of bacterialgenomes from Softberry ( http://www.softberry.com/berry.phtml) using the following parameters and cutoffs: open reading frame size = 100 aa, Expectation = 1× 10 ^−10^. Predicted open reading frames were queried against COG and GenBank nonredundant (NR) databases. SSU and LSU rRNA genes were identified by blastn query against NR with expectation cutoffs of 1 × 10 ^−8^. tRNAs were identified using the program tRNAscan-SE 1.21 ( http://www.genetics.wustl.edu/eddy/tRNAscan-SE) set to the archaeal tRNA covariance model [ 75]. Automated FGENESB annotation of fosmids was manually refined and corrected using the genome annotation and visualization tool Artemis ( http://www.sanger.ac.uk/Software/Artemis) [ 76]. Identification of CO _2_ fixation and ammonia oxidation genes was based on the combined results of FGENESB output, manual annotation, and targeted tblastn queries against both GenBank, the environmental database (env_nt), and the set of seven sample bins containing individual WGS reads for the SAR. Putative crenarchaeal sequences identified in the set of SAR contigs assembled in env_nt on the basis of homology to specific open reading frames predicted in the C. symbiosum dataset were used to reciprocally identify individual WGS reads from the set of reads within each sample bin. A tblastn cutoff of 1 × 10 ^−40^ and percent identity >75% for protein coding genes and a blastn cutoff of 1 × 10 ^−90^ and percent identity >98% for SSU rRNA genes were used.

### Phylogenetic analysis

Comparative analysis was performed on *amoA, amoB,* and *amoC* sequences identified in this study and related sequences available in GenBank and the environmental database. In the case of *amoA,* sequences recovered by environmental library screening were compared to sequences identified in GenBank, the SAR, and the genome of C. symbiosum. In the case of *amoB* and *amoC,* only sequences identified in GenBank, the SAR, and the genome of C. symbiosum were compared. Deduced amino acid sequences for environmental *amoA* sequences were determined from 650 bp of overlapping nucleotide sequence. Protein alignments for all subunits were generated using the Clustal method. Phylogenetic trees for α, β, and γ subunits encoded by *amoA, amoB,* and *amoC,* respectively, were based on comparison of 191, 199, and 148 parsimony informative characters, respectively. Trees for all three subunits were generated using distance and parsimony methods implemented in PAUP* version 4.0b10 [ 77] and rooted with the corresponding subunit for particulate methane monooxygenase. Bootstrapping for distance and parsimony was accomplished with 1,000 replicates per tree using heuristic search methods. Maximum-likelihood tree reconstructions were performed with Tree-Puzzle v5.2 ( http://www.tree-puzzle.de) using a quartet puzzling procedure and 10,000 puzzling steps combined with a Mueller-Vingron model of substitution and a gamma-type distribution of rate heterogeneity with 16 categories. Bootstrap information was not included for a particular node if polytomy was observed using two or more tree reconstruction methods or if none of the bootstrap methods yielded a value of 70% or greater. Bootstrapping for maximum-likelihood was accomplished with 10,000 replicates.

## Supporting Information

Figure S1 Alignment of Deduced Amino Acid Sequences for Ammonia Monooxygenase Subunits Identified in C. symbiosum with Respect to Ammonia Monooxygenase and Particulate Methane Monooxygenase Subunits Identified in Environmental Libraries and Representative Archaeal and Bacterial Lineages
(A) α, (B) β, and (C) γ.
NITEU, *N. europaea,* and METTR, *Methylosinus trichosporium,* are used as primary reference sequences for ammonia monooxygenase and particulate methane monooxygenase subunits.
Dashes indicate missing residues or gaps. An asterisk indicates absolute conservation across all aligned sequences.
CENSY, *C. symbiosum;* NITMA, *Nitrosopumilus maritimus;* ACY, SAR; Soil, terrestrial soil sequence.
(7.9 MB PDF)

Figure S2 Alignment of Deduced Amino Acid Sequences for nirK Identified in C. symbiosum with Respect to Nitrite Reductase Identified in Environmental Libraries and Representative Archaeal and Bacterial Lineages
NITEU, N. europaea is used as primary reference.
Dashes indicate missing residues or gaps. An asterisk indicates absolute conservation across all aligned sequences.
CENSY, *C. symbiosum,* AACY, SAR; Soil, terrestrial soil derived sequence; FRAsp, *Frankia sp.* CcI3; ALCsp, *Alcaligenes sp.* STC1; PSUsp, *Pseudomonas sp.* G-179; HALDE, Haloferax denitrificans.
(8.6 KB PDF)

Figure S3 Distribution of SAR WGS Reads Covering *acc, birA, amo,* and *ure* Genes Homologous to *C. symbiosum,* and the Distribution of Crenarchaeal SSU rRNA Gene Sequences from Individual Sample Bins
(1.8 KB PDF)

Table S1 C. symbiosum Fosmid Locus Index
(35 KB PDF)

Table S2 Putative Planktonic Crenarchaeal Contigs from SAR Identified on the Basis of Homology to Predicted 3-Hydroxypropionate Cycle Genes Identified on C. symbiosum Fosmids
(25 KB PDF)

Table S3 Putative Planktonic Crenarchaeal Contigs from SAR Identified on the Basis of Homology to Predicted Nitrogen Cycle Genes Identified on C. symbiosum Fosmids
(29 KB PDF)

### Accession Numbers

Ammonia monoxygenase subunit A sequences (accession numbers DQ333400–DQ333425) have been submitted to GenBank ( http://www.ncbi.nlm.nih.gov/Genbank, as have annotated C. symbiosum fosmid sequences (accession numbers DQ397540–DQ397640 and DQ397827–DQ397878).

## Abbreviations

BAC: bacterial artificial chromosome
bp: base pair
CoA: coenzyme A
H4F: tetrahydrofolate-dependent
H4MPT: tetrahydromethanopterin-dependent
Mb: million base pair
SAR: Sargasso Sea
TCA: tricarboxylic acid
WGS: whole genome shotgun
