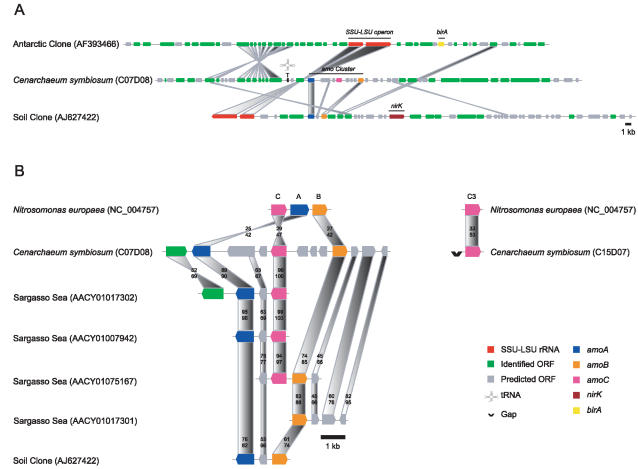# Correction: Pathways of Carbon Assimilation and Ammonia Oxidation Suggested by Environmental Genomic Analyses of Marine Crenarchaeota

**DOI:** 10.1371/journal.pbio.0040437

**Published:** 2006-12-12

**Authors:** Edward DeLong, Steven Hallam, Tracy Mincer, Christa Schleper, Christina Preston, Katie Roberts, Paul Richardson

In *PLoS Biology*, volume 4, issue 4: DOI: 10.1371/journal.pbio.0040095

There were errors in Figure 4. In (A), the shaded box connecting the hypothetical protein adjacent to the amoB subunit of C. symbiosum to a conserved homolog identified in the crenarchaeal soil clone (AJ627422) was misplaced. This box should be drawn from the C-terminal region of the larger open reading frame immediately upstream of the amoA subunit of C. symbiosum.

In (B), the N. europaea amo subunits were mislabeled and misplaced. Their orientation should be reversed such that the amoC subunit appears in opposite orientation to the far left, followed by the A subunit and then the B subunit. This results in the inversion of the shaded boxes connecting the N. europaea and C. symbiosum amo subunits with respect to the original image. The color coding of the unlinked amoC3 subunits is also incorrect. They should appear as pink colored boxes.

In the figure legend for both (A) and (B), the amoB and amoC sunbunits were inadvertently juxtaposed. The amoC subunit is represented by the pink colored box and the amoB subunit is represented by the orange colored box.

The corrected Figure 4 is attached.

**Figure:**