# Supplementary material for: Pathways of Carbon Assimilation and Ammonia Oxidation Suggested by Environmental Genomic Analyses of Marine Crenarchaeota
Source: PLoS Biol. 2006 Mar 21;4(4):e95. doi: 10.1371/journal.pbio.0040095 (PMC1403158; doi:10.1371/journal.pbio.0040095)
Supplement: Supplementary file 1 [file pbio.0040095.sg001.pdf]

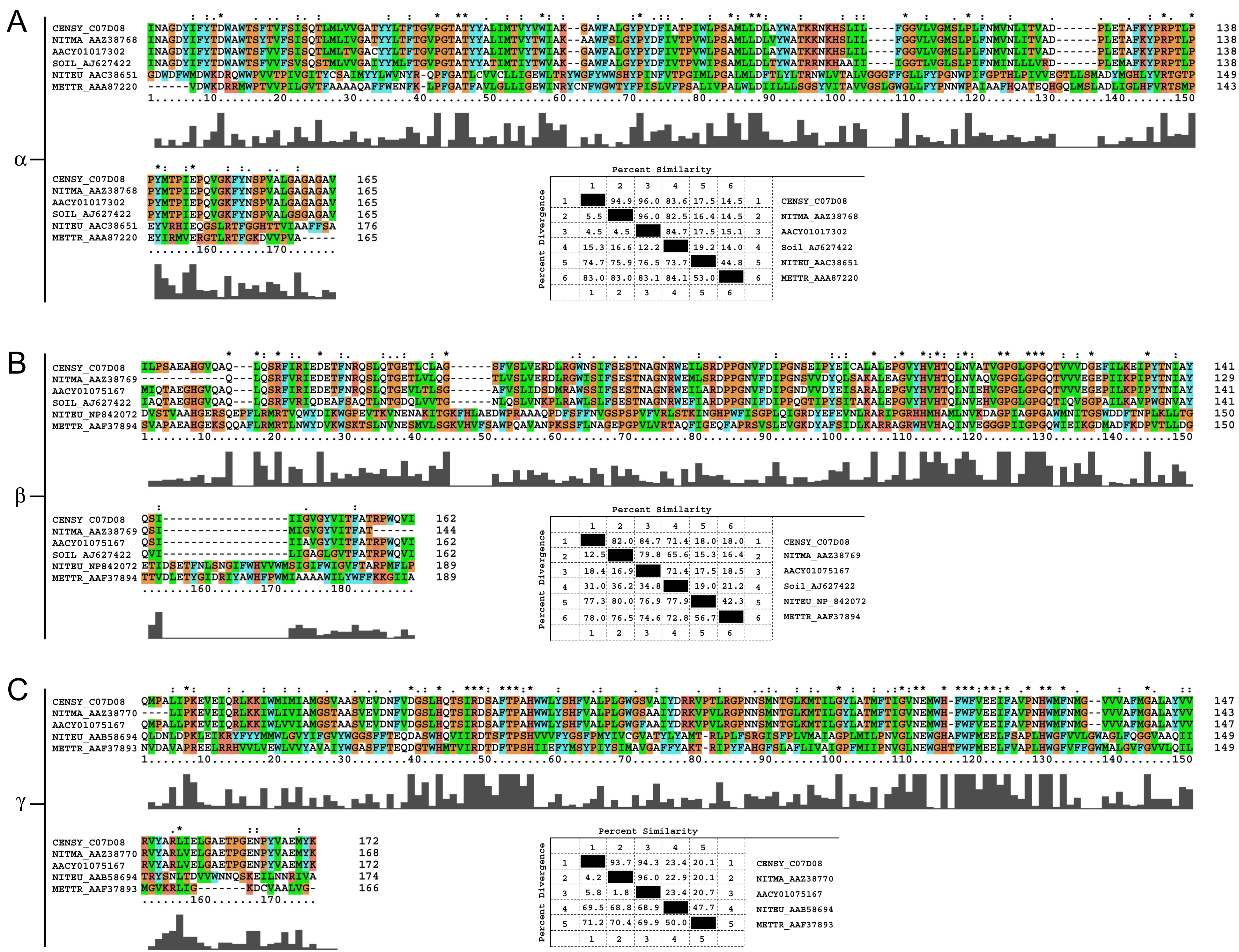

A
α
CENSY_C07D08
NITMA_AAZ38768
AACY01017302
SOIL_AJ627422
NITEU_AAC38651
METTR_AAA87220
Percent Similarity
Percent Divergence
1 94.9 96.0 83.6 17.5 14.5 1 CENSY_C07D08
2 5.5 96.0 82.5 16.4 14.5 2 NITMA_AAZ38768
3 4.5 4.5 84.7 17.5 15.1 3 AACY01017302
4 15.3 16.6 12.2 19.2 14.0 4 Soil_AJ627422
5 74.7 75.9 76.5 73.7 44.8 5 NITEU_AAC38651
6 83.0 83.0 83.1 84.1 53.0 6 METTR_AAA87220
B
β
CENSY_C07D08
NITMA_AAZ38769
AACY01075167
SOIL_AJ627422
NITEU_NP842072
METTR_AAF37894
Percent Similarity
Percent Divergence
1 82.0 84.7 71.4 18.0 18.0 1 CENSY_C07D08
2 12.5 79.8 65.6 15.3 16.4 2 NITMA_AAZ38769
3 18.4 16.9 71.4 17.5 18.5 3 AACY01075167
4 31.0 36.2 34.8 19.0 21.2 4 Soil_AJ627422
5 77.3 80.0 76.9 77.9 42.3 5 NITEU_NP_842072
6 78.0 76.5 74.6 72.8 56.7 6 METTR_AAF37894
C
γ
CENSY_C07D08
NITMA_AAZ38770
AACY01075167
NITEU_AAB58694
METTR_AAF37893
Percent Similarity
Percent Divergence
1 93.7 94.3 23.4 20.1 1 CENSY_C07D08
2 4.2 96.0 22.9 20.1 2 NITMA_AAZ38770
3 5.8 1.8 23.4 20.7 3 AACY01075167
4 69.5 68.8 68.9 47.7 4 NITEU_AAB58694
5 71.2 70.4 69.9 50.0 5 METTR_AAF37893
