# Supplementary material for: Pathways of Carbon Assimilation and Ammonia Oxidation Suggested by Environmental Genomic Analyses of Marine Crenarchaeota
Source: PLoS Biol. 2006 Mar 21;4(4):e95. doi: 10.1371/journal.pbio.0040095 (PMC1403158; doi:10.1371/journal.pbio.0040095)
Supplement: Supplementary file 2 [file pbio.0040095.sg002.pdf]

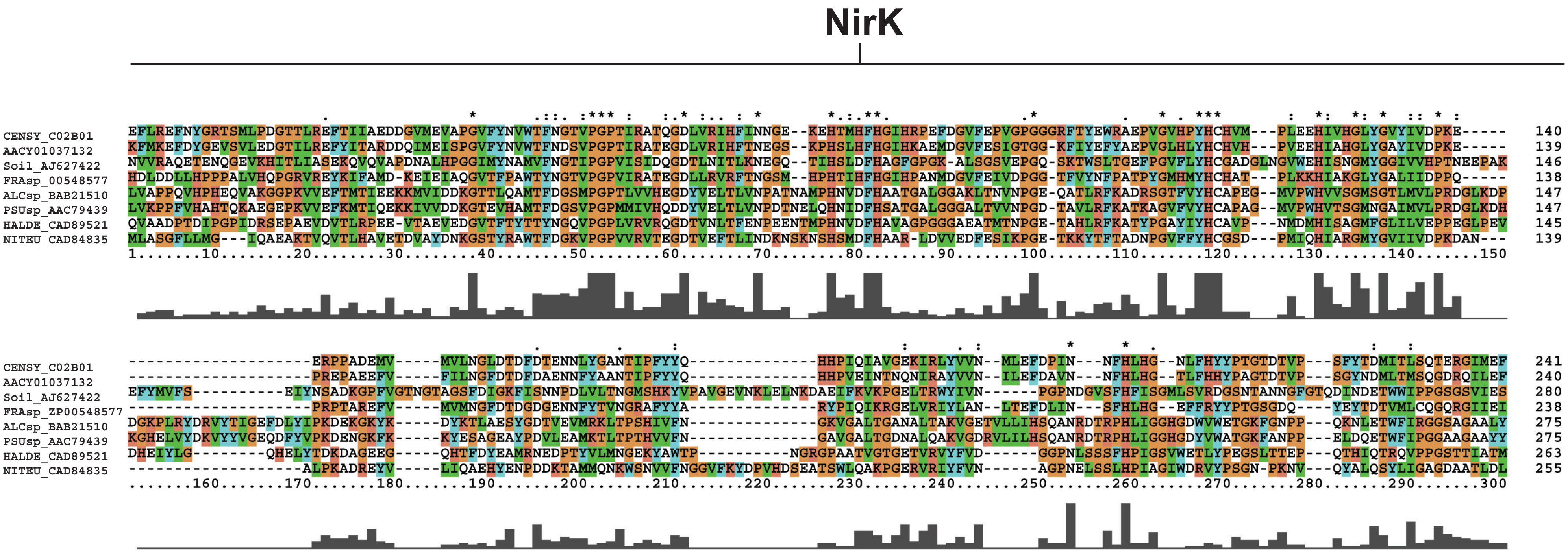

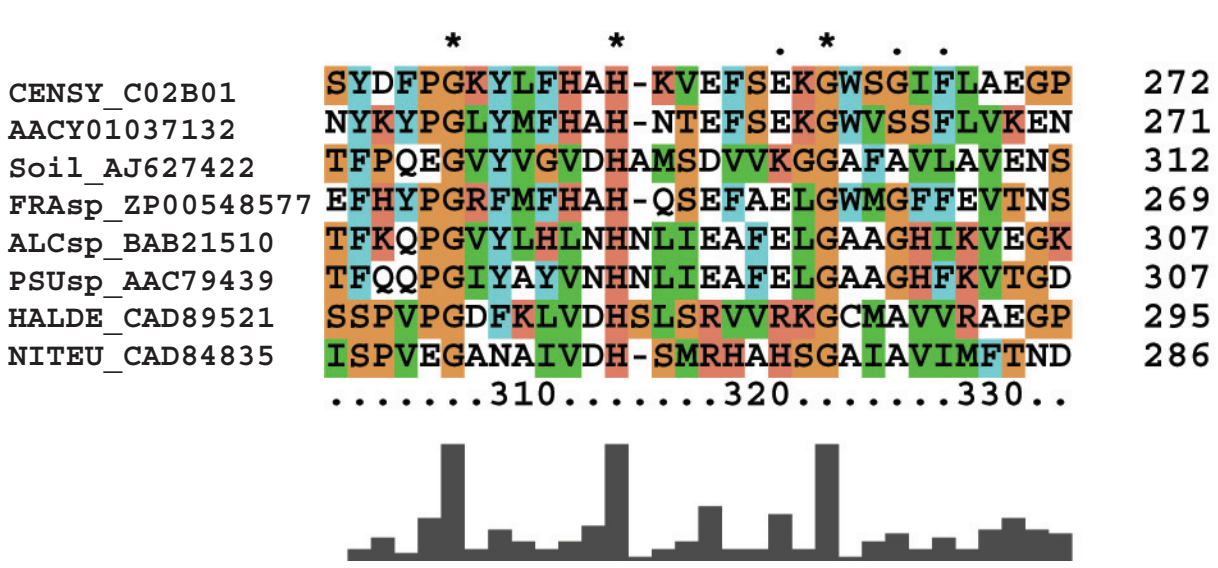

Percent Similarity (upper triangle) / Percent Divergence (lower triangle)

	1	2	3	4	5	6	7	8		
1		73.5	22.3	58.7	24.1	22.9	29.2	30.1	1	CENSY_C02B01
2	42.1		21.4	57.5	24.4	24.4	25.9	29.8	2	AACY01037132
3	77.7	80.2		23.2	19.9	18.7	29.8	28.6	3	Soil_AJ627422
4	54.5	52.7	74.3		25.0	26.2	29.5	31.6	4	FRASP_ZP00548577
5	77.1	79.9	75.8	76.4		72.0	31.0	25.6	5	ALCSP_BAB21510
6	79.4	79.7	76.3	74.3	29.5		29.8	25.6	6	PSUSP_AAC79439
7	75.4	78.1	70.1	72.5	68.3	70.5		33.4	7	HALDE_CAD89521
8	75.1	76.7	70.5	73.1	70.8	70.5	66.4		8	NITEU_CAD84835
	1	2	3	4	5	6	7	8		
