# Supplementary material for: Pathways of Carbon Assimilation and Ammonia Oxidation Suggested by Environmental Genomic Analyses of Marine Crenarchaeota
Source: PLoS Biol. 2006 Mar 21;4(4):e95. doi: 10.1371/journal.pbio.0040095 (PMC1403158; doi:10.1371/journal.pbio.0040095)
Supplement: Supplementary file 3 [file pbio.0040095.sg003.pdf]

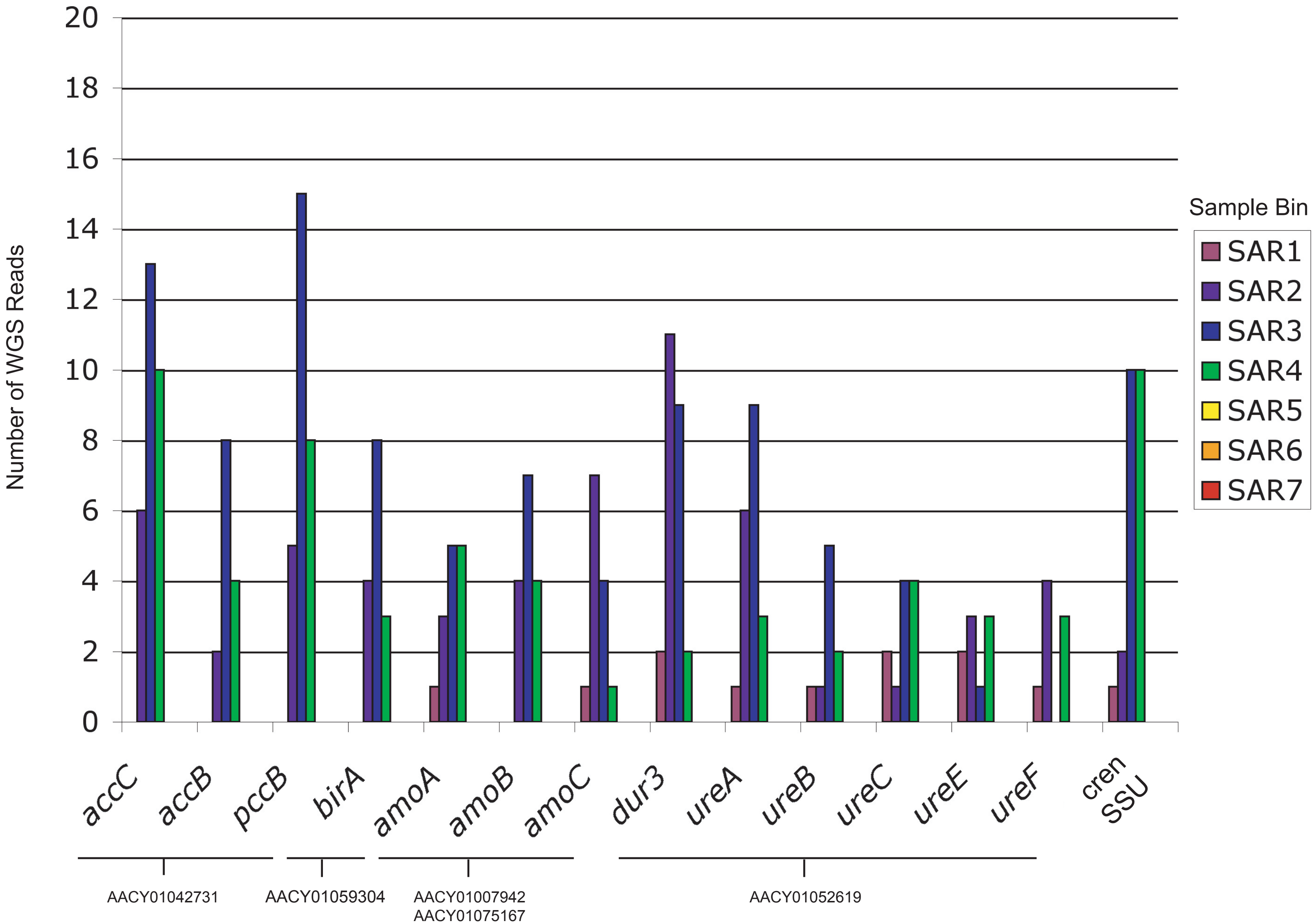

Number of WGS Reads
20
18
16
14
12
10
8
6
4
2
0
accC
accB
pccB
birA
amoA
amoB
amoC
dur3
ureA
ureB
ureC
ureE
ureF
cren SSU
AACY01042731
AACY01059304
AACY01007942
AACY01075167
AACY01052619
Sample Bin
SAR1
SAR2
SAR3
SAR4
SAR5
SAR6
SAR7
