# Supplementary material for: Pathways of Carbon Assimilation and Ammonia Oxidation Suggested by Environmental Genomic Analyses of Marine Crenarchaeota
Source: PLoS Biol. 2006 Mar 21;4(4):e95. doi: 10.1371/journal.pbio.0040095 (PMC1403158; doi:10.1371/journal.pbio.0040095)
Supplement: Supplementary file 4 [file pbio.0040095.st001.pdf]

Table S1 *Cenarchaeum symbiosum* fosmid locus index

					Pathway components*																																													
		Contig			3-hydroxypropionate cycle											TCA cycle																Ammonia oxidation and denitrification																		
cenFOS	JGI	1	2	G+C	*accC*	*accB*	*pccB*	*adh*	*ald*	*fadD*	*fadB*	*fadH*	*mce*	*mcm*	*birA*	*citZ*	*citE*	*acn*	*icd*	*oorA*	*oorB*	*sucD*	*sucC*	*sdhA*	*sdhB*	*sdhC*	*sdhD*	*fumC*	*mdh*	*ppsA*	*pckA*	*amt*	*dur3*	*ureA*	*ureB*	*ureC*	*ureE*	*ureF*	*ureG*	*ureH*	*amoA*	*amoB*	*amoC*	*azu*	*Fe-S*	*cyt*	*nirK*	*nirA*	*norD*	*norQ*
C01A01	3436580	37585		0.610																																														
C01A05	3436581	39291		0.594																									1																		1			
C01A08	3436582	41419		0.590																1	1										1																			
C01B03	3436583	43871		0.583																												1																		
C01B01	3436584	40703		0.607																																								3						
C01C01	3436585	41173		0.543						1																																		1	1	1				
C01C06	3436586	41548		0.586							1																						1	1	1	1	1	1	1	1										
C01C10	3436587	40511		0.612																																														
C02A01	3436588	33294		0.592																																														
C02B01	3436590	45105		0.592																									1																		1			
C02C04	3436591	42768		0.565																																														
C02C07	3436592	39120		0.600																																														
C02E10	3436593	42767		0.565																																														
C02E12	3436594	39848		0.588				1																																				1						
C02G05	3436595	44971		0.609																																								3						
C02H06	3436596	39848		0.588				1																																				1						
C03A05	3436597	39899		0.590				1	1																																									
C03G11	3436598	41322		0.549																																														
C03H05	3436599	39721		0.496																																								1	1	1				
C04E08	3436600	40224		0.627											1																																			
C04F04	3436601	26157		0.877								1					1																																	
C04G08	3436602	44564		0.590	1	1	1																																											
C04H09	3436603	41052	42804	0.572				1	1																																									
C05D01	3436605	37104		0.581				1																																										
C05D09	3436606	43893		0.593																																														
C06A09	3436607	38485		0.598																																														
C07C04	3436608	41652		0.544																		1	1																										1	1
C07G05	3436609	39781		0.548																																								1						
C07E08	3436610	40832		0.598																																														
C08F03	3436611	36219		0.584																																														
C09C10	3436612	39564		0.589																																														
C13A10	3436613	40870		0.604																																														
C15C12	3436614	41214		0.554																																														
C18C03	3436615	38829		0.511																																														
C18C04	3436616	36161		0.481																																														
C17D04	3436617	43042		0.597																1	1																													
C17E03	3436618	37670		0.520						1																							1	1	1	1								1	1	1				
C19H10	3436619	43787		0.551																																														
C21G07	3436620	38150		0.598																																														
C01A04	3634001	40230		0.600																																														
C01B07	3634002	41876		0.584																																														
C01D10	3634003	39454		0.588																									1																		1			
C01F05	3634004	40941		0.563																																														
C05C02	3634005	42705		0.589								1					1																											3						
C05D06	3634006	42904		0.585								1																																						
C05D08	3634007	39634		0.592																																														
C05G03	3634008	45533		0.568																																														
C06D08	3634009	38715		0.570																											1																	2		
C07B06	3634010	41389		0.582				1																																										
C07D04	3634011	41538		0.576																																														
C07D08	3634012	43141		0.583							1																														1	1	1							
C07E03	3634013	42905		0.611																								1																						
C07F11	3634014	34352		0.763																																														
C08D01	3634015	44495		0.457																																														
C08E01	3634016	37303		0.649				1																																				1						
C08E06	3634017	40636		0.566																																								2						
C09A12	3634018	43887		0.580									1	1					1											1																				
C09B07	3634019	40824		0.620				1																																										
C09C06	3634020	39895		0.601																																								2						
C09E09	3634021	42577		0.601																																														
C11D05	3634023	41867		0.602				1																																				1						
C13A11	3634024	40499		0.503																																								1	1	1				
C13D11	3634025	38832		0.543																																														
C13E07	3634026	38880		0.525						1	1																																							
C13E12	3634027	39464		0.577																																														
C13G08	3634028	38845		0.608								1					1																											1						
C15D08	3634029	38754		0.620				1																																										
C15E09	3634030	43895		0.596																									1																		1			
C16D03	3634031	42367		0.576																																														
C18D02	3634032	38412		0.582																																					1	1	1							
C18E05	3634033	39931		0.517																																														
C18H11	3634035	41492		0.581				1				1																																						
C19A05	3634036	40370		0.540																																														
C19E09	3634037	41893		0.573															1																															
C19G04	3634038	46841		0.604																																														
C20E06	3634039	45538		0.563														1				1	1																										1	1
C20G06	3634040	39443		0.565																																														
C20G08	3634041	40803		0.573																																														
C21A03	3634042	38527		0.577						1																																		1						
C21H07	3634044	38934		0.555														1																																
C05B02	3634045	39977		0.583																1	1																													
C01F04	3634552	37889		0.592																																														
C01F06	3634553	34540		0.606																																								1						
C01G02	3634554	39399		0.601																																								1						
C01G08	3634555	41923		0.600																																														
C03A04	3634556	41463		0.582																																							1							
C03B02	3634558	38692		0.570																																														
C03E10	3634559	39960		0.571																																														
C03F09	3634560	39172		0.589																												1												1						
C04D12	3634561	40123		0.505																																														
C05E01	3634562	42625		0.583																																														
C05G02	3634563	39265		0.569																1	1										1																			
C05G09	3634564	38695		0.539																																								1						
C06E05	3634565	42858		0.606																																														
C06H12	3634566	37698		0.596																																														
C07B08	3634567	41295		0.599				1																																										
C07D01	3634568	43795		0.601																																														
C07D05	3634569	43888		0.545						1																																			1	1				
C07E09	3634570	42614		0.612	1	1	1																	1	1	1	1																							
C07F02	3634571	41724		0.529																																														
C08B03	3634573	40416		0.518																		1	1																										1	1
C08B04	3634574	46177		0.600																																								3						
C08G10	3634575	37232		0.732																																														
C09D08	3634577	25122	11079	0.568																											1																	2		
C09D12	3634578	39299		0.493																																									1	1				
C09E11	3634579	41244		0.607											1																																			
C12A09	3634580	38974		0.597																																														
C12B02	3634581	36797		0.596																																														
C13E02	3634582	43826		0.579																		1									1																	2		
C14A09	3634583	45665		0.597																																														
C15A07	3634584	43680		0.603																																														
C15H04	3634587	44579		0.591																																														
C17B05	3634588	40924		0.574																												1																		
C19D08	3634591	40691		0.601																								1																						
C20A12	3634592	41539		0.568																																														
C20B09	3634593	39122		0.594																				1	1																									
C20C07	3634594	42246		0.587																																														
C21B03	3634595	40572		0.609																																														
C21F02	3634596	39981		0.617																				1	1	1	1																							
C21G04	3634597	36742		0.594																																														
C03C07	4001018	41693		0.508																																														
C11A07	4001019	38449		0.600				1																																										
C07H12	4001020	40298		0.513																																														
C21E11	4001021	41768		0.572																																														
C19D04	4001022	42161		0.518																																														
C15D07	4001023	43122		0.537																																							1	1						
C13A04	4001024	38317		0.545																																														
C20B06	4001025	45783		0.559												1																																		
C16G08	4001026	39063		0.505																																														
C02B01	4001027	40010		0.572								1																				1												1						
C05A02	4001028	37577		0.582																																														
C03F10	4001029	41348		0.585														1																																
C12D02	4001030	38783		0.565																											1																	2		
C21C08	4001032	37513		0.597																																														
C101G10	na	32998		0.555				1	1																																									
C98C02	na	40000		0.574																																														
C01B12	na	34772		0.573																																														
C04A07	na	42553		0.580								1																				1												1						
C04G09	na	33298		0.601																								1																						
C05E04	na	40921		0.558																																														
C05H05	na	43064		0.556																																														
C16A06	na	33861		0.499																																									1	1				
C16E09	na	40387		0.598																				1	1	1	1																							
C04E12	na	40810		0.512																																														
C04C05	na	40287		0.580																																														
C04G10	na	40996		0.616																																														
C60A05	na	42432		0.586																																														
147		5983105		0.579	2	2	2	14	3	5	3	7	1	1	2	1	3	3	2	4	4	4	3	4	4	3	3	3	4	1	6	5	2	2	2	2	1	1	1	1	2	2	4	34	7	7	4	8	3	3

homolog (green)

conserved domain (yellow)

* Pathway component abbreviations for 3-hydroxypropionate cycle: acc = acetyl-CoA carboxylase, adh = alcohol dehydrogenase, ald = aldehyde dehydrogenase, fadD = acyl-CoA synthetase, fadB = enoyl-CoA hydratase, fadH = enoyl-CoA reductase, mce = methylmalonyl-CoA epimerase, mcm = methylmalonyl-CoA mutase, birA = biotin ligase. TCA cycle citZ = citrate synthase, citE = citrate lyase, citryl-CoA lyase, acn = aconitase, icd = isocitrate dehydrogenase, oor = 2-oxoacid ferredoxin oxidoreductase, suc = succinyl-CoA synthetase, sdh = succinate dehydrogenase/fumarate reductase, fumC = fumarate hydratase, fumarase, mdh = malate/lactate dehydrogenase, ppsA = phosphoenolepyruvate synthase, pckA = phosphoenolepyruvate carboxylase. Ammonia oxidation and denitrification: amt = amonia permease, dur = urea transporter, ure = urease, amo = ammonia monooxygenase, azu = azurin/plastocyanin, Fe-S = iron-sulfur cluster, cyt = cytochrome, nirA ferredoxin nitrite/sulfite reductase, nirK = nitrite reductase, nor = nitric oxide reductase
