# Supplementary material for: Pathways of Carbon Assimilation and Ammonia Oxidation Suggested by Environmental Genomic Analyses of Marine Crenarchaeota
Source: PLoS Biol. 2006 Mar 21;4(4):e95. doi: 10.1371/journal.pbio.0040095 (PMC1403158; doi:10.1371/journal.pbio.0040095)
Supplement: Supplementary file 5 [file pbio.0040095.st002.pdf]

Table S2 Putative planktonic crenarchaeal contigs from Sargasso Sea identified on the basis of homology to predicted *C. symbiosum* genes

	env_nt	bp	3-hydroxypropionate cycle components^															
			accC	*accB*	*pccB*	*adh*	*ald*	*fadD*	*fadB*	*fadH*	*mce*	*mcm*	*birA*	*sdhA*	*sdhB*	*sdhC*	*sdhD*	*fumC*
1	AACY01042731.1	9339	1	1	1													
2	AACY01063159.1	1808	1		1													
3	AACY01149055.1	923		1														
4	AACY01150397.1	846		1														
5	AACY01531440.1	936		1														
6	AACY01595481.1	815		1														
7	AACY01016865.1	1375			1													
8	AACY01523534.1	829			1													
9	AACY01003323.1	1414				1												
10	AACY01006139.1	1440				1	1											
11	AACY01020457.1	19487				1	1		1									
12	AACY01073689.1	2008				1												
13	AACY01079528.1	2763				1												
14	AACY01116472.1	1644				1	1											
15	AACY01158405.1	1032				1												
16	AACY01393097.1	795				1												
17	AACY01526616.1	921				1												
18	AACY01550717.1	657				1												
19	AACY01580464.1	886				1												
20	AACY01583337.1	877				1												
21	AACY01624844.1	880				1	1											
22	AACY01788972.1	847				1												
23	AACY01079534.1	25743						1	1									
24	AACY01109620.1	3182						1	1									
25	AACY01017014.1	1848							1									
26	AACY01020458.1	1378							1									
27	AACY01020459.1	9443							1									
28	AACY01143215.1	1116							1									
29	AACY01192926.1	849							1									
30	AACY01418071.1	823							1									
31	AACY01535186.1	930							1									
32	AACY01566646.1	775							1									
33	AACY01625968.1	838							1									
34	AACY01661864.1	876							1									
35	AACY01792017.1	898							1									
36	AACY01020307.1	12432								1								
37	AACY01023385.1	1303								1								
38	AACY01053432.1	2024								1								
39	AACY01073141.1	4477								1								
40	AACY01088240.1	2272								1								
41	AACY01149604.1	986								1								
42	AACY01159254.1	1015								1								
43	AACY01443705.1	818								1								
44	AACY01472497.1	653								1								
45	AACY01474905.1	868								1								
46	AACY01485167.1	798								1								
47	AACY01521069.1	898								1								
48	AACY01535564.1	694								1								
49	AACY01546378.1	810								1								
50	AACY01560127.1	706								1								
51	AACY01571442.1	898								1								
52	AACY01573143.1	880								1								
53	AACY01578058.1	908								1								
54	AACY01589619.1	962								1								
55	AACY01598863.1	825								1								
56	AACY01032962.1	2599									1							
57	AACY01052621.1	8163									1	1						
58	AACY01100445.1	1091									1							
59	AACY01265496.1	804									1							
60	AACY01452825.1	928									1							
61	AACY01633190.1	767									1							
62	AACY01019350.1	2697										1						
63	AACY01032961.1	1862										1						
64	AACY01068289.1	3260										1						
65	AACY01109991.1	6869										1						
66	AACY01431384.1	754										1						
67	AACY01512631.1	931										1						
68	AACY01593487.1	880										1						
69	AACY01629095.1	772										1						
70	AACY01059304.1	2446											1					
71	AACY01093101.1	6732											1					
72	AACY01107362.1	1393											1					
73	AACY01308462.1	992											1					
74	AACY01353735.1	864											1					
75	AACY01595191.1	939											1					
76	AACY01722930.1	918											1					
77	AACY01081100.1	6126												1	1	1	1	
78	AACY01058001.1	4701												1		1		
79	AACY01537877.1	840												1		1		
80	AACY01143154.1	938												1				
81	AACY01508886.1	898												1				
82	AACY01541593.1	933												1				
83	AACY01543587.1	944												1				
84	AACY01165736.1	953												1				
85	AACY01549419.1	697												1				
86	AACY01473655.1	912												1			1	
87	AACY01042729.1	1467												1				
88	AACY01042750.1	2892													1			
89	AACY01058002.1	2198													1	1	1	
90	AACY01153209.1	858													1			
91	AACY01042730.1	8437													1	1	1	
92	AACY01301021.1	899													1			
93	AACY01311915.1	981														1	1	
94	AACY01345013.1	832														1		
95	AACY01705852.1	718														1		
96	AACY01609527.1	893															1	
97	AACY01201460.1	902															1	
98	AACY01079537.1	5416																1
99	AACY01118725.1	3172																1
100	AACY01050699.1	1458																1
101	AACY01301827.1	976																1
102	AACY01059123.1	1556																1
103	AACY01453165.1	797																1
104	AACY01145310.1	934																1
105	AACY01780012.1	840																1
		237277	2	5	4	14	4	2	14	20	6	9	7	11	6	8	7	8

(green) indicates conservation of two or more genes adjacent to the *C. symbiosum* gene of interest spanning more than 2 kb

1 indicates positive identification of a *C. symbiosum* homologue

^ identification based on tblastn results contrained to E-values > E-10, amino acid identity > 55% and word sizes greater than 100 amino acids

env_nt = environmental data base

Component abbreviations for 3-hydroxypropionate pathway: 3-hydroxypropionate cycle: acc = acetyl-CoA carboxylase, adh = alcohol dehydrogenase, ald = aldehyde dehydrogenase, fadD = acyl-CoA synthetase, fadB = enoyl-CoA hydratase, fadH = enoyl-CoA reductase, mce = methylmalonyl-CoA epimerase, mcm = methylmalonyl-CoA mutase, birA = biotin ligase, sdh = succinate dehydrogenase/fumerate reductase, fumC = fumarate hydratase, fumarase
