# Supplementary material for: Pathways of Carbon Assimilation and Ammonia Oxidation Suggested by Environmental Genomic Analyses of Marine Crenarchaeota
Source: PLoS Biol. 2006 Mar 21;4(4):e95. doi: 10.1371/journal.pbio.0040095 (PMC1403158; doi:10.1371/journal.pbio.0040095)
Supplement: Supplementary file 6 [file pbio.0040095.st003.pdf]

Table S3 Putative planktonic crenarchaeal contigs from Sargasso Sea identified on the basis of homology to predicted *C. symbiosum* genes

			Ammonia oxidation and denitrification pathway components^															
	env_nt	bp	*amt*	*dur3*	*ureA*	*ureB*	*ureC*	*ureE*	*ureF*	*ureG*	*ureH*	*amoA*	*amoB*	*amoC*	*azu*	*nirK*	*norD*	*norQ*
1	AACY01005312.1	2448	1															
2	AACY01063826.1	2249	1															
3	AACY01152142.1	985	1															
4	AACY01805534.1	980	1															
5	AACY01095574.1	4929	1															
6	AACY01345107.1	731	1															
7	AACY01095572.1	11014	1															
8	AACY01315813.1	709	1															
9	AACY01329653.1	555	1															
10	AACY01701463.1	852	1															
11	AACY01038588.1	1623	1															
12	AACY01071834.1	2659	1															
13	AACY01145875.1	971	1															
14	AACY01032963.1	2816		1														
15	AACY01052619.1	7415		1	1	1	1	1	1									
16	AACY01052621.1	8163		1														
17	AACY01072213.1	1004		1														
18	AACY01117804.1	1825		1														
19	AACY01133314.1	791		1														
20	AACY01265585.1	862		1														
21	AACY01320484.1	884		1														
22	AACY01397955.1	809		1														
23	AACY01476530.1	698		1														
24	AACY01553196.1	670		1														
25	AACY01557965.1	778		1														
26	AACY01567355.1	909		1														
27	AACY01605005.1	756		1														
28	AACY01627393.1	907		1														
29	AACY01020806.1	1159			1													
30	AACY01032964.1	1572			1													
31	AACY01354563.1	787			1													
32	AACY01400051.1	908			1													
33	AACY01564278.1	851			1													
34	AACY01712486.1	971			1													
35	AACY01007887.1	1772				1	1	1	1									
36	AACY01067332.1	1552				1	1	1										
38	AACY01117805.1	1338				1	1	1										
39	AACY01476699.1	919				1												
41	AACY01540570.1	482				1												
42	AACY01605004.1	674				1												
43	AACY01000023.1	2839						1	1	1	1							
44	AACY01329189.1	862						1	1									
45	AACY01485679.1	822						1	1									
46	AACY01557964.1	769						1	1									
47	AACY01733346.1	859						1	1									
48	AACY01229883.1	933							1									
49	AACY01679717.1	818							1	1								
50	AACY01688298.1	921							1									
51	AACY01552106.1	535								1								
52	AACY01093526.1	1706									1							
53	AACY01109210.1	1064									1							
54	AACY01202733.1	897									1							
55	AACY01397954.1	759									1							
56	AACY01497609.1	949									1							
57	AACY01007942.1	1514										1		1				
58	AACY01017302.1	2995										1		1				
59	AACY01020737.1	2430										1						
60	AACY01057256.1	1174										1		1				
61	AACY01075168.1	1436										1						
62	AACY01435967.1	914										1						
63	AACY01500052.1	784										1						
64	AACY01575171.1	914										1						
65	AACY01606230.1	873										1						
66	AACY01765291.1	803										1						
67	AACY01017301.1	2213											1					
68	AACY01075167.1	2250											1	1				
69	AACY01204262.1	963											1	1				
72	AACY01500051.1	946											1					
74	AACY01571827.1	759											1					
76	AACY01666890.1	865											1					
77	AACY01679565.1	818											1					
78	AACY01330706.1	914												1				
79	AACY01788167.1	323												1				
80	AACY01002884.1	3317													1			
81	AACY01004940.1	1378													1			
82	AACY01008003.1	1505													1			
83	AACY01010871.1	4763													1			
84	AACY01021537.1	1377													1			
85	AACY01021538.1	3114													1			
86	AACY01053215.1	1439													1			
87	AACY01071841.1	2410													1			
88	AACY01084931.1	6284													1			
89	AACY01092260.1	8437													1			
90	AACY01095570.1	1314													1			
91	AACY01135033.1	837													1			
92	AACY01163974.1	907													1			
93	AACY01287819.1	844													1			
94	AACY01352818.1	902													1			
95	AACY01358573.1	859													1			
96	AACY01369416.1	729													1			
97	AACY01403436.1	905													1			
98	AACY01409077.1	803													1			
99	AACY01448863.1	841													1			
100	AACY01470382.1	905													1			
101	AACY01486130.1	847													1			
102	AACY01489585.1	935													1			
103	AACY01489612.1	859													1			
104	AACY01495157.1	900													1			
105	AACY01506902.1	937													1			
106	AACY01513539.1	815													1			
107	AACY01516366.1	856													1			
108	AACY01522489.1	936													1			
109	AACY01532452.1	952													1			
110	AACY01539091.1	866													1			
111	AACY01539708.1	783													1			
112	AACY01550291.1	578													1			
113	AACY01561692.1	828													1			
114	AACY01572635.1	906													1			
115	AACY01592642.1	935													1			
116	AACY01602681.1	872													1			
117	AACY01607693.1	711													1			
118	AACY01611932.1	895													1			
119	AACY01612696.1	846													1			
120	AACY01626428.1	879													1			
121	AACY01669880.1	882													1			
122	AACY01679734.1	887													1			
123	AACY01693823.1	983													1			
124	AACY01715516.1	890													1			
125	AACY01745401.1	911													1			
126	AACY01788189.1	846													1			
127	AACY01021539.1	3643														1		
128	AACY01026733.1	1195														1		
129	AACY01037132.1	2219														1		
130	AACY01080607.1	1538														1		
131	AACY01098185.1	1781														1		
132	AACY01513526.1	781														1		
133	AACY01604695.1	794														1		
134	AACY01607692.1	894														1		
135	AACY01612697.1	882														1		
136	AACY01625324.1	955														1		
137	AACY01771368.1	916														1		
138	AACY01001989.1	4236															1	1
139	AACY01036144.1	1440															1	
140	AACY01075165.1	13276															1	1
141	AACY01172916.1	985															1	
142	AACY01499482.1	885															1	
143	AACY01530245.1	839															1	
144	AACY01018267.1	3801																1
145	AACY01641481.1	883																1
146	AACY01001990.1	1568																1
		221514	13	15	7	7	4	9	10	3	6	10	7	7	47	11	6	5

(green) indicates conservation of two or more genes adjacent to the *C. symbiosum* gene of interest spanning more than 2 kb

1 indicates positive identification of a *C. symbiosum* homologue

^ identification based on tblastn results contrained to E-values > E-10, amino acid identity > 55% and word sizes greater than 100 amino acids

env_nt = environmental data base

Component abbreviations for ammonia oxidation and denitrification pathway: amt = amonia permease, dur = urea transporter, ure = urease, amo = ammonia monooxygenase, azu = azurin/plastocyanin, nirK = nitrite reductase, nor = nitric oxide reductase
